# Coupling the Antimalarial Cell Penetrating Peptide TP10 to Classical Antimalarial Drugs Primaquine and Chloroquine Produces Strongly Hemolytic Conjugates

**DOI:** 10.3390/molecules24244559

**Published:** 2019-12-12

**Authors:** Luísa Aguiar, Arnau Biosca, Elena Lantero, Jiri Gut, Nuno Vale, Philip J. Rosenthal, Fátima Nogueira, David Andreu, Xavier Fernàndez-Busquets, Paula Gomes

**Affiliations:** 1 LAQV-REQUIMTE, Departamento de Química e Bioquímica, Faculdade de Ciências, Universidade do Porto, 4169-007 Porto, Portugal; luisa.aguiarts@gmail.com; 2 Barcelona Institute for Global Health (ISGlobal, Hospital Clínic-Universitat de Barcelona), Rosselló 149-153, 08036 Barcelona, Spain; abiosca@ibecbarcelona.eu (A.B.); elantero@ibecbarcelona.eu (E.L.); xfernandez_busquets@ub.edu (X.F.-B.); 3 Nanomalaria Group, Institute for Bioengineering of Catalonia (IBEC), The Barcelona Institute of Science and Technology, Baldiri Reixac 10-12, 08028 Barcelona, Spain; 4 School of Medicine, University of California at San Francisco, 1001 Potrero Avenue, San Francisco, San Francisco, CA 94110, USA; jiri.gut@ucsf.edu (J.G.); Philip.Rosenthal@ucsf.edu (P.J.R.); 5 Departamento de Farmacologia, Departamento de Ciências do Medicamento, Faculdade de Farmácia da Universidade do Porto, Rua de Jorge Viterbo Ferreira 228, 4050-313 Porto, Portugal; nuno.vale@ff.up.pt; 6 IPATIMUP—Instituto de Patologia e Imunologia Molecular da Universidade do Porto, Rua Júlio Amaral de Carvalho 45, 4200-135 Porto, Portugal; 7 i3S, Instituto de Investigação e Inovação em Saúde, Rua Alfredo Allen 208, 4200-135 Porto, Portugal; 8 Global Health and Tropical Medicine, Instituto de Higiene e Medicina Tropical, Universidade Nova de Lisboa, 1349-008 Lisbon, Portugal; FNogueira@ihmt.unl.pt; 9 Department of Experimental and Health Sciences, Pompeu Fabra University, Barcelona Biomedical Research Park, Dr. Aiguader 88, 08003 Barcelona, Spain; david.andreu@upf.edu; 10 Nanoscience and Nanotechnology Institute (IN2UB), University of Barcelona, Martí i Franquès 1, 08028 Barcelona, Spain

**Keywords:** antimalarial, cell penetrating peptide, chloroquine, erythrocyte fluorescence, flow cytometry, hemolysis, microscopy, *Plasmodium*, primaquine, red blood cell

## Abstract

Recently, we disclosed primaquine cell penetrating peptide conjugates that were more potent than parent primaquine against liver stage *Plasmodium* parasites and non-toxic to hepatocytes. The same strategy was now applied to the blood-stage antimalarial chloroquine, using a wide set of peptides, including TP10, a cell penetrating peptide with intrinsic antiplasmodial activity. Chloroquine-TP10 conjugates displaying higher antiplasmodial activity than the parent TP10 peptide were identified, at the cost of an increased hemolytic activity, which was further confirmed for their primaquine analogues. Fluorescence microscopy and flow cytometry suggest that these drug-peptide conjugates strongly bind, and likely destroy, erythrocyte membranes. Taken together, the results herein reported put forward that coupling antimalarial aminoquinolines to cell penetrating peptides delivers hemolytic conjugates. Hence, despite their widely reported advantages as carriers for many different types of cargo, from small drugs to biomacromolecules, cell penetrating peptides seem unsuitable for safe intracellular delivery of antimalarial aminoquinolines due to hemolysis issues. This highlights the relevance of paying attention to hemolytic effects of cell penetrating peptide-drug conjugates.

## 1. Introduction

**Figure 1:**
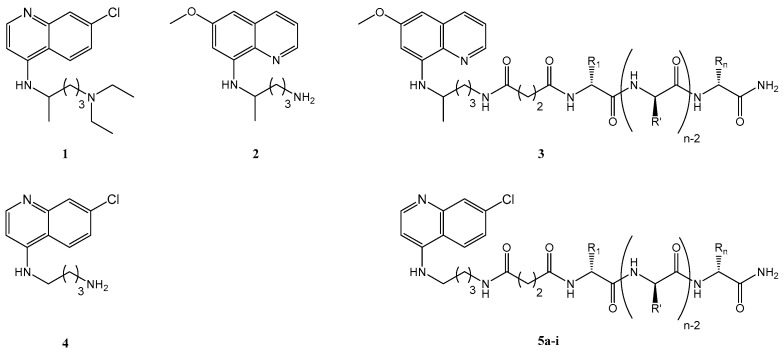
Structures of CQ (**1**), PQ (**2**), PQ- CPP conjugates previously disclosed by us (**3**) [20], and the chloroquine analogue (Cq, **4**) bearing a free primary amine to be linked to CPP (Table 1), and afford the first generation of Cq-CPP conjugates (Cq-C4-CPP, **5a-i**).

Malaria is responsible for about half a million deaths every year and represents a great threat for the billions of people living in or traveling to endemic areas. This parasitic disease is caused by different species of the *Plasmodium* genus, in particular *P. falciparum* and *P. vivax*. The life cycle of malaria parasite within the human body is complex, but can be divided in two stages: (i) the asymptomatic liver stage, which progresses to (ii) the blood stage, when clinical illness appears [1]. Apart from the complexity of the *Plasmodium* life cycle, several other factors hinder the eradication of this disease, such as (i) widespread resistance to cheaper and safer antimalarial drugs like chloroquine (CQ, **1** in Figure 1), (ii) toxicity of many older antimalarials, e.g., primaquine (PQ, **2** in Figure 1), quinacrine (CQR) or amodiaquine, (iii) low oral bioavailability, e.g., PQ, (iv) lack of effective vaccines, and (v) scarcity of multi-stage antimalarials that are able to efficiently eliminate liver- and blood-stage forms of *Plasmodium* parasites [2]. Resistance to newer antimalarials, including artemisinin-based combination therapies that are standard to treat CQ-resistant *falciparum* malaria has also emerged. Therefore, the search for new antimalarial therapeutic strategies remains an urgent task.

One of our main lines of research concerns the chemical modification of classical antimalarials such as PQ, CQ, or CQR, aimed at “masking” the drugs in such a way that their activity is preserved or even improved, while undesirable metabolic conversions and/or toxicity are decreased, and/or parasite resistance pathways are eluded [3,4,5,6,7,8,9,10]. Recently, we hypothesized that coupling cell-penetrating peptides (CPP) to classical antimalarials could both improve internalization into *Plasmodium*-infected cells and avoid resistance and pharmacokinetic liabilities. CPP have been widely explored as targeting agents for several small molecules, as they can efficiently permeate cellular membranes without requiring specific receptors or compromising membrane integrity, while carrying a wide range of cargoes, from proteins to nucleic acids, inside living cells [11].

While CPP have been widely investigated mostly for cancer or genetic disorders [12,13,14,15], their use as targeting agents for parasitic diseases, including malaria, is yet underexplored, although the combination (either ionic or covalent) of fosmidomycin to the octaarginine CPP has been proven a successful strategy to improve the antiplasmodial activity of this antibiotic. [16]. The relevance of filling this void is clear if we consider the potential value of previously reported antimalarial peptides, such as (i) TP10, a CPP with activity against blood-stage malaria infection [17], (ii) DPT-sh1 and DPT-sh2, reported to preferentially internalize *Plasmodium*-infected red blood cells (PiRBC) over healthy erythrocytes (hRBC) [18], or (iii) IDR-1018, a broad-spectrum antimicrobial and immunomodulatory peptide [19]. In view of this, we recently explored how conjugation of the antimalarial drug PQ with selected CPP would influence its action against liver-stage *Plasmodium* parasites, and found that activity was improved in PQ-CPP conjugates (**3** in Figure 1) with TP10 or the related peptide Transportan as the CPP moiety [20]. Building on these findings, and considering that reports on antimalarial activity of TP10 and other peptides, like DPT-sh1 and -sh2, addressed blood-stage infections, we have now investigated whether conjugation to CPP would be equally promising when applied to CQ, the classical blood-stage antimalarial that is currently limited by widespread drug resistance [21].

## 2. Results

### 2.1. Synthesis and Antiplasmodial Activity of First-Generation Drug-Peptide Conjugates **5a**–**5i**

**Scheme 1:**
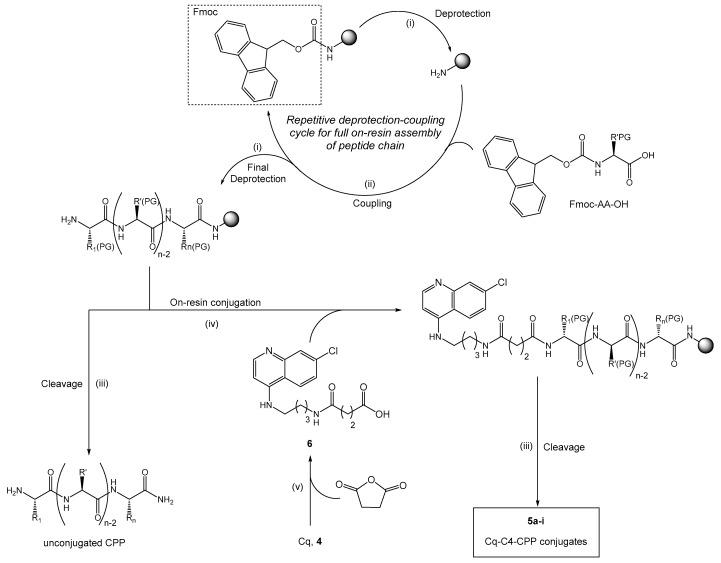
Synthesis of conjugates **5a**-**5i**: (i) 20% piperidine in dimethylformamide (DMF) containing 0.1 M of 1-hydroxybenzotriazole (HOBt), microwave irradiation (MWI) for 30 s at 24 W plus 3 min at 28 W; (ii) 5 eq, respective to the resin reactive groups, of the Fmoc-AA-OH in DMF (0.2 M), 5 eq of 0.5 M *O*-(Benzotriazol-1-yl)-*N,N,N′,N*′-tetramethyluronium hexafluorophosphate (HBTU)/HOBt in DMF, and 10 eq of 2 M *N*-ethyl-*N*,*N*-diisopropylamine (DIPEA) in *N*-methylpyrrolidone (NMP), MWI for 5 min at 35 W; (iii) trifluoroacetic acid (TFA)/water/triisopropylsilane (TIS) 95:2.5:2.5 *v*/*v*/*v* or TFA/thioanisole/ethane-1,2-dithiol/anisole 90:5:3:2 *v*/*v*/*v*/*v* cocktail (1 mL/100 mg of resin), room temperature (RT), 120 min; (iv) 5 eq of **6** and of (benzotriazol-1-yloxy)tripyrrolidinophosphonium hexafluorophosphate (PyBOP), 10 eq DIPEA in DMF, RT, overnight; (v) 1.2 eq of succinic anhydride and of DIPEA in DMF, RT, 120 min. Grey sphere stands for the Rink-amide resin used as polymer support used for solid-phase peptide synthesis (SPPS). PG, protecting group.

Drug-CPP conjugates **5a–5i** (Figure 1), derived from chloroquine analogue **4** (Cq, Figure 1) and based on analogous PQ-CPP conjugates **3**, were successfully prepared through a combination of solution- and solid-phase synthesis procedures (Scheme 1).

All conjugates and their respective parent CPP (Table 1), were screened in vitro for their antiplasmodial activity, using the CQ-resistant *P. falciparum* W2 strain. The primaquine analogue to **5a**, i.e., conjugate **3** where the CPP is TP10, PQ-C4-TP10 (**3a**), was also included in the study for comparison.

Results (Table 2) showed that, unlike previously found for PQ-CPP conjugates **3** (Figure 1) [20], none of their CQ counterparts **5a-i** displayed an activity equal or superior to that of the commercial reference drug, CQ (**1**, IC_50_ = 699 nM) [22]. Still, these data cannot be directly compared, as they refer to different species (*P. berghei* vs. *P. falciparum*) and developmental stages (liver vs. blood) of malaria parasites. Nonetheless, the activity of the PQ-C4-TP10 conjugate **3a** against blood-stage *P. falciparum* W2 was also evaluated, but no consistent data could be obtained, due to apparently strong hemolytic effects that were later confirmed (*cf.* Section 2.2. and Table 3). Regarding unmodified CPP, all but TP10 (IC_50_ = 5.53 µM) and Transportan (IC_50_ = 3.05 µM), which are closely related to each other (TP10 is an *N*-terminally truncated derivative of Transportan; see their shared sequence underlined in Table 1), were inactive (IC_50_ > 10 µM) against *P. falciparum*. This was not surprising, since TP10 has been shown to possess intrinsic antiplasmodial activity [8], whereas the other unrelated CPP have not.

Only three of the Cq-C4-CPP conjugates, namely, **5a**, **5b,** and **5g**, displayed IC_50_ values below 10 µM, with TP10- and Transportan-derived conjugates **5a** (IC_50_ = 1.52 µM) and **5b** (IC_50_ = 5.20 µM) being the most active. Interestingly, while conjugation of Cq to Transportan affording conjugate **5b** caused a decrease in antiplasmodial activity, the opposite occurred in the TP10-derived conjugate, **5a**, for which antiplasmodial activity was enhanced over that of the parent CPP. Still, the activity of this conjugate was lower than that of the parent drug, CQ.

These results clearly show that antiplasmodial activity of the conjugates is influenced by both the aminoquinoline and the peptide building blocks. The lack of activity of DPT-sh1 and -sh2 derived conjugates, **5c** and **5d**, was somewhat surprising, as those peptides had been reported to preferentially internalize PiRBC [18]. Considering these results, we hypothesized that the modest or absent antiplasmodial activity of conjugates **5a**-**5i** might be explained by inadequate linkage between CPP and drug, as linker features such as length, stability, lipophilicity, or lability towards specific environments are likely to affect the overall performance of the conjugate [14,23,24].

### 2.2. Synthesis, Antiplasmodial, and Hemolytic Activity of Second-Generation Conjugates **10–14**

To test the above hypothesis, i.e., to explore how variation of the spacer and conjugation chemistry might affect the antiplasmodial activity of Cq-CPP conjugates, and keeping in mind that conjugation of Cq to TP10 produced the most active first-generation conjugate, **5a**, a series of novel conjugates (Table 3) was synthesized, with the aminoquinoline linked to TP10 through different spacers, namely, a larger flexible alkyl spacer (Cq-C10-TP10, **10**), to increase the lipophilicity and drug’s exposure; a rigid 1,2,3-triazole ring (Cq-TR-TP10, **11**), which has been widely explored for the modification of antiplasmodial agents [25,26,27,28], and a disulfide linker (Cq-S-S-TP10, **12**), providing a labile (chemo- and bio-reducible) linkage between peptide and drug. Moreover, to evaluate the relevance of the relative position of drug and peptide building blocks (drug coupled at the peptide *N*- or *C*-terminus), two additional conjugates were synthesized, with Cq conjugated to the TP10 *C*-terminal region through a succinyl (TP10-C4-Cq, **13**) or disulfide (TP10-S-S-Cq, **14**) linker.

**Figure 2:**
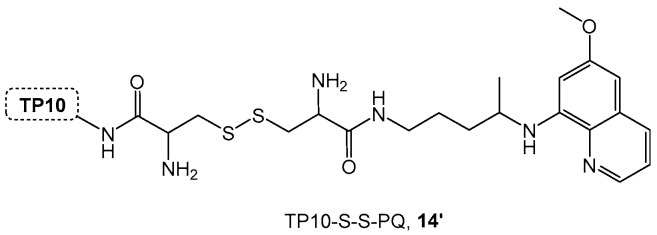
Structure of conjugate **14′**, TP10-S-S-PQ.

**Scheme 2:**
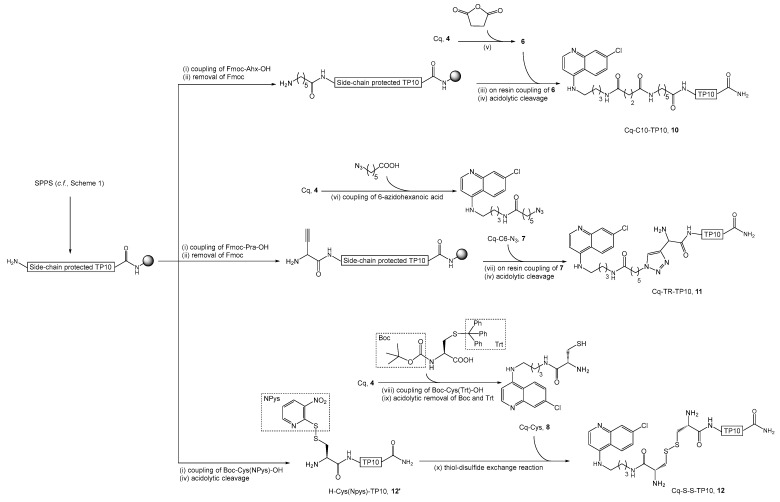
Synthesis of conjugates **10–12**: (i) 5 eq, respective to the resin reactive groups, of the fluorenylmethyloxycarbonyl (Fmoc) or tert-butyloxycarbonyl (Boc)-AA-OH in DMF (0.2 M), 5 eq of 0.5 M HBTU/HOBt in DMF, and 10 eq of 2 M DIPEA in *N*-NMP, MWI for 5 min at 35 W; (ii) 20% piperidine in DMF containing 0.1 M of HOBt, MWI for 30 s at 24 W plus 3 min at 28 W; (iii) 5 eq of **6** and of PyBOP, 10 eq DIPEA in DMF, RT, overnight; (iv) TFA/water/TIS 95:2.5:2.5 *v*/*v*/*v* (1 mL/100 mg of resin), RT, 120 min; (v) 1.2 eq of succinic anhydride and of DIPEA in DMF, RT, 120 min; (vi) 6-azido hexanoic acid (1.2 eq), HBTU (1.2 eq), DIPEA (2.4 eq) in DMF, 0 °C → RT, 3 h; (vii) copper(I) bromide (1.2 eq), 2,6-lutidine (10 eq), sodium ascorbate (3 eq), and DIPEA (10 eq), solubilized in a mixture of acetonitrile (ACN) and DMF (1:2 *v*/*v*), RT, overnight; (viii) Boc-Cys(Trt)-OH (1.2 eq), HBTU (1.2 eq), DIPEA (2.4 eq) in DMF, 0 °C → RT, 3 h; (ix) TFA:TIS:H_2_O 95:2.5:2.5 (*v*/*v*/*v*), 1 h, 0 °C → RT; (x) 1 M aqueous acetic acid, RT, 36 h. Grey sphere stands for the Rink-amide resin used as polymer support used for SPPS. Ph, phenyl group.

**Scheme 3:**
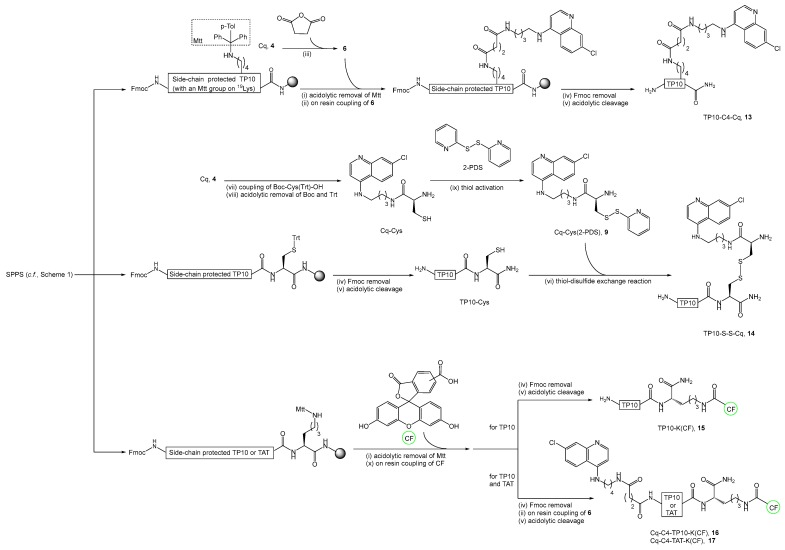
Synthesis of conjugates **13–17**: (i) resin treated with 1% TFA in DCM, RT, until no yellow color detected in filtrate; (ii) 5 eq of **6** and of PyBOP, 10 eq DIPEA in DMF, RT, overnight; (iii) 1.2 eq of succinic anhydride and of DIPEA in DMF, RT, 120 min; (iv) 20% piperidine in DMF containing 0.1 M of HOBt, MWI for 30 s at 24 W plus 3 min at 28 W; (v) TFA/water/TIS 95:2.5:2.5 *v*/*v*/*v* (1 mL/100 mg of resin), RT, 120 min; (vi) 1 M aqueous acetic acid, RT, 36 h; (vii) Boc-Cys(Trt)-OH (1.2 eq), HBTU (1.2 eq), DIPEA (2.4 eq) in DMF, 0 °C → RT, 3 h; (viii) TFA:TIS:H_2_O 95:2.5:2.5 (*v*/*v*/*v*), 1 h, 0 °C → RT; (ix) 2,2′-dithiopyridine (2-PDS, 2.0 eq), glacial acetic acid (0.3 eq), anhydrous MeOH, 18 h, RT; (x) 5(6)-carboxyfluorescein (CF; 5.0 eq), PyBOP (5.0 eq), DIPEA (10 eq), DMF, 2 h, RT.

These second-generation conjugates (synthesis routes depicted in Scheme 2; Scheme 3) were evaluated in vitro, alongside the commercial reference drug, CQ (**1**), their parent building blocks, TP10 and Cq (**4**), and first-generation conjugate **5a**, regarding their ability to inhibit growth of blood-stage parasites of the CQ-sensitive *P. falciparum* 3D7 strain (Table 3). The primaquine analogues of Cq conjugates **5a** and **14**, respectively, PQ-C4-TP10 (**3a**) and TP10-S-S-PQ (Figure 2, **14′**), were also included for comparison; the synthesis of these conjugates, **3a** and **14′**, has been reported elsewhere [20]. Of note, substantial hemolysis was detected upon erythrocyte exposure to some of the test conjugates, particularly at the higher concentrations tested. In view of this, emolysis assays were also conducted on cultures of both hRBC and PiRBC (Table 3). 

Antiplasmodial activities of CQ (**1**), TP10 and **5a** were greater against the CQ-sensitive 3D7 strain than against the CQ-resistant W2 strain (Table 2 and Table 3). While increased activity against CQ-sensitive 3D7 versus CQ-resistant W2 was expected for CQ, these results highlight that resistance mechanisms in *P. falciparum* W2 also alter its sensitivity to the unmodified peptide and its Cq conjugate. The W2 strain is resistant to many antimalarials, including CQ, and has been reported to exhibit multiple resistance determinants [29], including mutations in the *P. falciparum* CQ resistance transporter (PfCRT) and *P. falciparum* multi-drug resistance transporter 1 (PfMDR1) [25]. In fact, parasite resistance to CQ has been primarily associated with mutations in PfCRT [30,31], for which mechanisms mediating resistance are not well understood, but which likely include changes in the pH of the parasite digestive vacuole (DV), limiting accumulation of basic CQ [32,33]. Hence, it is reasonable to assume that, as they are also basic, both the CPP and its conjugate **5a** will have lower accumulation in the DV of W2, compared to 3D7 parasites.

The in vitro activity of Cq (**4**), which was the building block actually used in the synthesis of conjugates **5** and **10–14**, was comparable to that of CQ (**1**); this was an expected observation, based on the well-known structure-activity relationships described for CQ and related compounds, which highlight the vital role of the 7-chloro-4-aminoquinoline core and the possibility to carry out slight to moderate changes in the aliphatic moiety without a major impact on activity [34]. Moreover, the equimolar mixture of Cq (**4**) with TP10 was also tested, but the apparent IC_50_ value displayed, which was similar to that of Cq alone, cannot be regarded as an accurate one, as high standard deviation values were obtained. Still, this suggests that there are no synergistic effects arising from non-covalent combination of those two building blocks.

Regarding the influence of the linker and relative position of the peptide and drug building blocks on antiplasmodial activity, comparison of IC_50_ values obtained for conjugates **5a** and **10–14** allow us to infer that: (i) increasing the size and, consequently, hydrophobicity, of an alkyl spacer (**5a** versus **10**) does not significantly alter activity; (ii) increasing polarity and rigidity of the linker (as in **11** versus **5a** or **10**) causes a decrease in antiplasmodial activity; (iii) replacing a stable linker (as in **5a**, **10** or **11**) by a chemo- and bio-reversible disulfide, as in **12**, does not contribute to an increase in antiplasmodial activity; (iv) altering the order in which peptide and drug are linked together, i.e., moving the antimalarial building block from an *N*-terminal (as in **12**) to a *C*-terminal (as in **14**) position does not favor antiplasmodial activity.

Relevantly, for PQ-derived conjugates **3a** and **14′**, i.e., where the 4-aminoquinoline building block in **5a** and in **14**, respectively, was replaced by the antimalarial 8-aminoquinoline PQ, in vitro data on blood-stage activity could not be reliably determined, due to the extensive hemolytic effects displayed by those conjugates (Table 3). Hemolytic activity was equally considerable for Cq-derived conjugates **5a** and **10–14**, and data compiled on Table 3 show that: (i) while CQ (**1**) and its analogue Cq (**4**) are not hemolytic, and peptide TP10 is only slightly hemolytic, all conjugates are significantly hemolytic, especially for cultures exclusively containing hRBC; (ii) the least hemolytic conjugate is **12**, where Cq is linked to TP10 through a bioreducible disulfide bridge, indicating that (iii) using a stable linker as in **5a, 10,** and **11** significantly increases hemolysis, which might be associated with a “detergent”-like action on erythrocyte membranes; (iv) coupling the drug to the peptide *C*- rather than *N*-terminus augments hemolytic activity, as illustrated by data for **14** vs. **12** and for **13** vs. **5a**.

In fact, the significant hemolytic activity of **13** impeded the determination of consistent antiplasmodial activity data, as occurred with the PQ-derived conjugates **3a** and **14′**; (v) % hemolysis was greater for erythrocyte cultures exclusively containing hRBC than for those where 3% of the erythrocytes were PiRBC, suggesting that the conjugates are more disruptive to membranes of hRBC than to those of PiRBC.

Still in regard to hemolysis, the equimolar mixture of Cq and TP10 displayed very low hemolytic activity, similar to that of TP10 alone; this highlights the fact that the substantial hemolytic effects observed for drug-peptide conjugates only arise when both building blocks are covalently bound to each other. Noteworthy, the hemolytic activity previously reported for Transportan [35] differs from that found by us for TP10; this is not surprising, as, though related, Transportan and TP10 are not the same peptide. Still, the hemolytic activity of TP10 has also been reported before [36], and differs from that obtained by us. However, in the previously reported study, whole blood samples treated with an anticoagulant were used for hemolysis assays, whereas we opted to use isolated erythrocytes, i.e., separated from the other blood components. This choice was based in the fact that fluorescently labeled analogues of our conjugates (see Section 2.3) were found to gather around, and internalize, leucocytes (data not shown); this precludes accurate determination of hemolysis that, by definition, is the destruction of red blood cells.

Considering the findings above described, further studies of interactions between Cq-CPP conjugates and erythrocytes (both hRBC and PiRBC) were pursued, by means of fluorescence microscopy and flow cytometry, as follows.

### 2.3. Insights into the Interactions between Chloroquine-Peptide Conjugates and Erythrocytes

Erythrocyte targeting and internalization studies were carried out using fluorescently labeled derivatives of the most active conjugate **5a** (**16**), of the parent peptide TP10 (**15**), and of the inactive conjugate Cq-C4-TAT **5f** (**17**), for comparison. Fluorescent labeling was achieved by coupling 5(6)-carboxyfluorescein (CF) to a lysine residue added to the *C*-terminus of the peptide sequence, as depicted in Scheme 3. After incubating the fluorescently labeled compounds **15–17** in asynchronous cultures of *P. falciparum* 3D7 at 3% parasitemia (i.e., containing 3% PiRBC and 97% hRBC), the nuclei of intraerythrocytic parasites were stained with DNA-binding Hoechst 33342 [37], and cell suspensions analyzed through flow cytometry.

**Figure 3:**
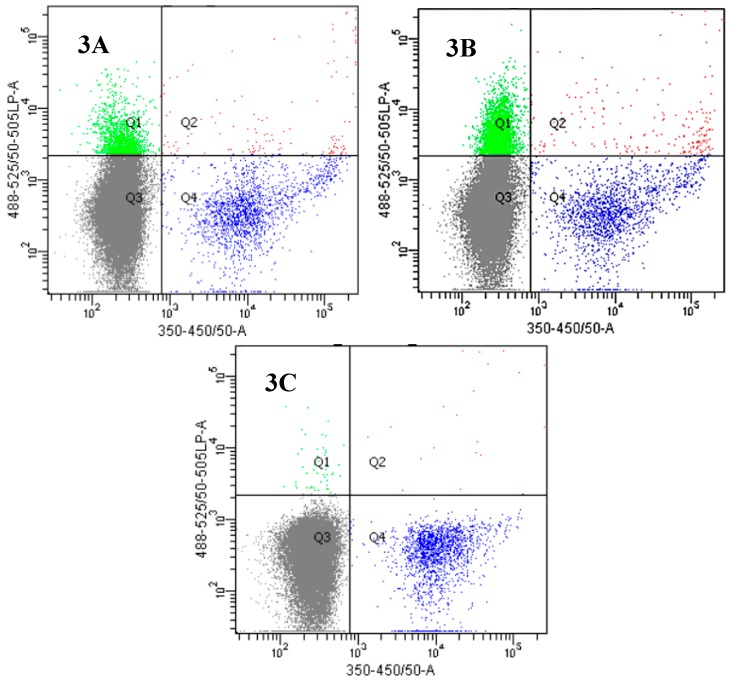
Flow cytometry analysis of the interaction between hRBC/PiRBC and CF-labelled (**A**) TP10, (**B**) Cq-C4-TP10, and (**C**) Cq-C4-TAT. Upper quadrants (Q1 and Q2) refer to compound-bound cells (green CF fluorescence) and right-hand quadrants (Q2 and Q4) refer to PiRBC (blue Hoechst fluorescence). Q3 includes hRBC, which were not internalized by the CF-labelled test compounds.

Quantitative data from this analysis (Figure 3), allowed us to conclude that: (i) binding of the fluorescently labeled Cq-C4-TP10 (**16**) to red blood cells (hRBC + PiRBC) is significantly higher than that of unconjugated TP10 (**15**), essentially due to much increased interaction of **16** with hRBC, as compared to the peptide, since the degree of binding of either peptide (**15**) or conjugate (**16**) to PiRBC does not substantially differ; and (ii) the interaction of fluorescently labeled Cq-C4-TAT (**17**) with either uninfected or parasitized RBC, is negligible.

**Figure 4:**
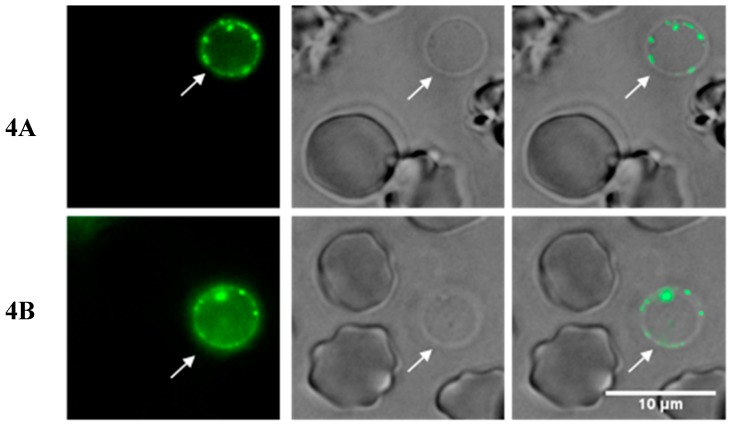
Microscopy fluorescence images showing the interaction between hRBC and (**A**) CF-labeled TP10 (**15**) and (**B**) CF-labeled Cq-C4-TP10 (**16**). Arrows depict “ghost” uninfected erythrocytes.

**Figure 5:**
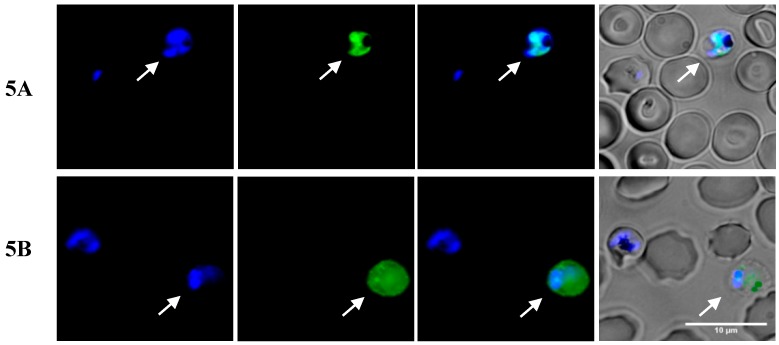
Microscopy fluorescence images showing the interaction between PiRBC (distinguished by the blue staining of parasites’ nuclei) and (**A**) CF-labeled TP10 (**15**) and (**B**) CF-labeled Cq-C4-TP10 (**16**). Arrows depict “ghost” *Plasmodium*-infected erythrocytes.

Quantitative analysis of cell suspensions was complemented by fluorescence microscopy studies. As shown in Figure 4A,B, both the CF-labeled peptide TP10 (**15**) and its Cq-conjugate **16** apparently interact only with RBC that show significant membrane damage, so-called “ghost” erythrocytes. In turn, no green fluorescence was detected on hRBC with an apparently intact membrane or in the presence of the inactive Cq-TAT conjugate **17** (not shown). Likewise, both labeled TP10 (**15**) and its Cq conjugate (**16**) seemed to interact preferentially with PiRBC (parasitized cells can be identified by blue staining parasites nuclei), which show clearly damaged membranes and host parasites that are at a later stage of development (Figure 5A,B).

**Figure 6:**
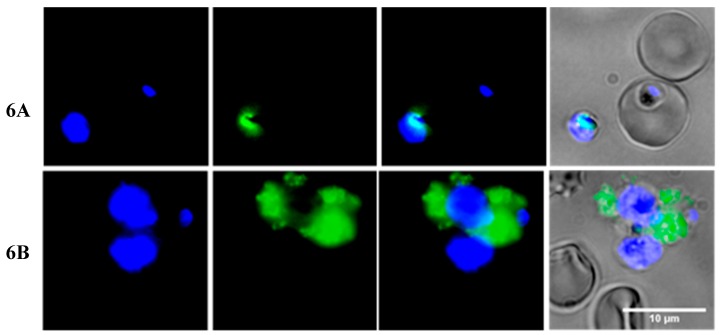
Microscopy fluorescence images showing an apparent co-localization of (**A**) CF-labeled TP10 (**15**) and DNA of parasites released after PiRBC disruption, or of (**B**) CF-labeled Cq-C4-TP10 (**16**) and what appears to be PiRBC debris surrounding nuclei of released parasites.

While unconjugated peptide (**15**) apparently co-localizes with parasite DNA (Figure 6A), its Cq conjugate **16** does not; instead, the latter seems to be bound to what appears to be PiRBC debris produced by cellular disruption upon parasite release (Figure 6B), although, without additional studies, one cannot rule out the hypothesis of being a lymphocyte phagocytizing the peptide. Taken together, these observations suggest that peptide and conjugate have different modes of interaction, with an apparently stronger perturbation of the host cell membrane by the conjugate, consistent with its greater hemolytic activity compared to the unconjugated peptide (Table 3).

Although it can be argued that insertion of an external moiety, like a fluorescent tag, onto a bioactive molecule, could dramatically change its physiological behavior, this concern is practically limited to cases where a large fluorophore is coupled to a small drug. However, many studies on the ability of distinct CPP and CPP conjugates to internalize different types of cells, including small constructs designed to cross the blood-brain barrier, have relied on the use of differently sized fluorophores, including very large ones, such as fluorescent proteins or quantum dots [38]. This implicitly means that such diverse fluorescent cargos do not considerably change the cell-penetration/interaction properties of the labelled peptide constructs. The same can be assumed in our case, since the distinct behavior of the fluorescently-labelled TP10 and TAT conjugates reflected the in vitro anti-plasmodial and hemolytic data on the respective untagged analogues. Indeed, the fact that both TP10 and its conjugate only interact with RBC whose membranes are visibly thinned agrees with in vitro data associating these compounds with hemolytic effects. None of the compounds were observed inside ring stage PiRBC, but only in hRBC (Figure 4) or PiRBC at later stages of parasite development (Figure 5). This suggests that, as soon as it invades an RBC, the parasite induces modifications in the membrane that render it more impermeable to both the CPP and its drug conjugate; later, as RBC egress is approached, increased permeability of both the parasitophorous vacuole and the erythrocyte membrane may facilitate internalization and subsequent antiplasmodial action [39]. Lastly, although it remains to be confirmed if interactions between the TP10-derived conjugate **16** and RBC membranes are the major cause for the display of antiplasmodial activity, it is undeniable that they must have a role in such activity, as they were not observed for its inactive TAT-derived analogue **17**.

## 3. Discussion

Taken together, our results demonstrate that significant changes in the mode of interaction of TP10, an antiplasmodial CPP, with RBC are induced upon conjugation of the peptide with the aminoquinoline Cq (4). However, Cq alone cannot account for the hemolytic and antiplasmodial properties of the conjugate, as Cq-C4-TAT did not display a similar behavior; instead, the latter was unable to invade erythrocytes or interact with their membranes, which is clearly correlated with its lack of antiplasmodial activity.

The significant increase in the hemolytic activity of TP10 upon conjugation to the 4-aminoquinoline suggests that drug cargo prevents an otherwise active CPP carrier from exerting the desired cell penetrating/antiplasmodial action safely, as it produces conjugates that exert membranolytic activity. Similarly, we recently found that PQ-CPP conjugates **3** (Figure 1) [20], including the PQ-TP10 conjugates **3a** and **14′**, respectively analogue to **5a** and **14**, are also significantly hemolytic (Table 3). Thus, hemolytic effects are seen with conjugation to a 4- or 8-aminoquinoline. Also, lytic effects might be specific to RBC, as PQ-CPP conjugate **3** is not toxic to hepatocytes [20]. RBC have specific characteristics, including being enucleated, having a highly plastic biconcave discoid structure, and stabilization by the so-called RBC membrane skeleton [40]. As such, membrane interaction and internalization phenomena in RBC are complex and different from those operating in other types of mammalian cells [41]. Moreover, it has been recently suggested that changes in membrane and membrane-skeleton organization continuously occur from the early stages of erythropoiesis up to the end of the life of an erythrocyte [42]. In addition, upon invasion by *Plasmodia*, erythrocyte membranes undergo several changes [43,44,45]. In any event, both this and previous reports [8] demonstrate that TP10 is a CPP able to (i) interact with PiRBC membranes, and (ii) display antiplasmodial activity, without provoking significant hemolysis. However, attaching aminoquinolines does not contribute to increase antiplasmodial activity, and triggers important hemolytic effects. Possibly, the heterocyclic aminoquinoline core inserts into the fatty acid chains of the RBC membrane lipid bilayer, causing an increase in the lipid-lipid distance and in membrane fluidity, ultimately leading to RBC disruption. This is being further studied using biophysical techniques.

## 4. Materials and Methods

### 4.1. Chemicals and Reagents

Except where otherwise stated, chemicals used for this work were purchased from Sigma-Aldrich. Rink-amide 4-methylbenzhydrylamine (MBHA) resin (0.52 mmol/g), *N*^α^-Fmoc or Boc-protected amino acids, and HBTU, for SPPS, were purchased from NovaBiochem. High performance liquid chromatography (HPLC) gradient or liquid chromatography-mass spectrometry (LC-MS) grade solvents were purchased from Merck.

Aluminum foils coated with silica-gel 60 F254 from Merck (Darmstadt, Germany) were used for thin-layer chromatography (TLC), and TLC chromatograms obtained were revealed under ultra-violet light (254 nm), using a Viber Lourmat lamp, model CN-6 (Vilber, Eberhardzell, Germany).

Purifications by liquid column chromatography were performed utilizing silica gel 60 as stationary phase. The mobile phase varied for each compound and is specified below.

*P. falciparum* 3D7 strain (chloroquine and mefloquine susceptible) was continuously cultured as previously described [46]. Parasitemia was monitored daily by microscopic examination of Giemsa (MERCK, Sigma-Aldrich) stained thin blood smears.

### 4.2. Instrumentation and General Procedures

Solid phase synthesis was carried out on a Liberty 1 microwave-assisted solid phase peptide synthesizer (CEM Corporation, Mathews, NC, USA). Peptide analysis by analytical high performance liquid chromatography (HPLC) was done using a Hitachi-Merck Elite LaChrom system from Hitachi High-Technologies America (Schaumburg, IL, USA) equipped with an L-2130 quaternary pump, an L-2200 thermostatted (Peltier effect) automated sampler, and an L-2455 diode-array detector (DAD). Samples were prepared by solubilization of crude products in aqueous acetic acid (10% *v*/*v*) and injected in a reverse phase C18 column Purospher star RP-C18 of 125 × 4.0 mm with 5 µm pore size. A gradient elution of varying proportions of 0.05% aqueous TFA (eluent A) and acetonitrile (ACN) (eluent B) was carried out for 30 min, at a flow rate of 1 mL/min, and detection was set at 220 nm. A Finnigan Surveyor LCQ DECA XP MAX spectrometer was used either alone, for direct electrospray ionization-ion trap mass spectrometry (ESI-IT MS) analysis, or coupled to a Finnigan Surveyor HPLC, equipped with a DAD Plus Detector, an Autosampler Plus and an LC Pump Plus, from ThermoElectron Corporation (Waltham, MA, USA), for LC-MS analysis. In either case, samples were prepared by sample solubilization in aqueous acetic acid (10% *v*/*v*). ESI-IT MS spectra and chromatograms obtained for the synthesized compounds can be found in Supplementary Materials.

Purifications of CPP and CPP-drug conjugates were carried out by preparative HPLC, to a purity of at least 90%. To this end, samples were injected in a LaPrep Sigma VWR preparative HPLC system equipped with an UV detector LP 3104 and an LP 1200 pump; a reverse-phase C18 column (250 × 25 mm ID with 5 µm pore size) from Merck was used, and a gradient elution using a varying relative proportion of eluent A and eluent B was carried out for 60 min at a flow rate of 15 mL/min. Pure peptide fractions were pooled and freeze-dried, using a VirTis freeze drier, model BenchTop Pro 9L with Omnitronics, from SP Scientific. A FACSort flow cytometer using CellQuest software (Becton, Dickinson, and Company, Franklin Lakes, NJ, USA) was used for antimalarial activity assays in vitro. For fluorescence microscopy studies, an Olympus IX51 inverted system microscope, equipped with an IX2-SFR X-Y stage, a U-TVIX-2 camera and a fluorescence mirror unit cassette for UV/blue/green excitation and detection of the corresponding blue/green/red emission ranges, was used. A BD LSRFortessa cell analyzer (Becton Dickinson S.A., San Agustín del Guadalix, Spain) was used for FCM.

### 4.3. Solution-Phase Synthesis

#### 4.3.1. Synthesis of *N*-(7-Methylquinolin-4-yl)butane-1,4-diamine, Cq (**4**)

We mixed 1.0 molar equivalents (eq) of 4,7-dichloroquinoline and 10.0 eq of butane-1,4-diamine in a round bottom flask, and the resulting solution was heated to 100 °C and left under reflux for 3 h. Then, the mixture was allowed to cool down to room temperature (RT), diluted with 25 mL of dichloromethane (DCM) and washed three times with an aqueous solution of Na_2_CO_3_. The organic phase was then dried with anhydrous Na_2_SO_4_ and, after filtration, the DCM was evaporated under reduced pressure. The resulting white solid (80% yield) was chromatographically homogeneous (TLC), and presented correct mass spectral data (ESI-IT MS, positive mode): m/z calculated for C_13_H_16_ClN_3_: 249.10 atomic mass units (a.m.u.); found: 250.33 (quasi-molecular ion, [M + H^+^]^+^).

#### 4.3.2. Synthesis of 3-({4-[(7-Methylquinolin-4-yl)amino]butyl}carbamoyl)propanoic acid, Cq-C4 (**6**)

Compound **4** (1.0 eq), succinic anhydride (1.2 eq), and DIPEA (1.2 eq) were dissolved in DMF, and the mixture was stirred for 2 h (RT). Then, the mixture was diluted with a 1:1 (*v*/*v*) mixture of DCM and methanol (MeOH), and purified by liquid chromatography on a silica gel column, using the same DCM/MeOH mixture as eluent. Compound **6** was obtained as a chromatographically homogeneous white solid (85% yield), whose structure was confirmed by ESI-IT MS (+). *m*/*z* calculated for C_17_H_20_ClN_3_O_3_: 349.12 a.m.u.; found: 350.67 ([M + H^+^]^+^).

#### 4.3.3. 6-Azido-*N*-{4-[(7-Methylquinolin-4-yl)amino]butyl}hexanamide, Cq-C6-N_3_ (**7**)

6-azido hexanoic acid (1.2 eq) was mixed with HBTU (1.2 eq), and DIPEA (2.4 eq), in DMF, and the mixture placed under stirring in an ice-water bath, for 30 min. Then, compound **4** (1.0 eq) was added, the reaction left under stirring in the ice-water bath, and its progress monitored by TLC. After 3 h, the mixture was diluted in ethyl acetate (20 mL) and washed with 5% aqueous Na_2_CO_3_ (3 × 15 mL). The organic layer was dried with anhydrous Na_2_SO_4_ and, after filtration, ethyl acetate was removed by evaporation under reduced pressure. The crude residue was then purified by liquid column chromatography on silica gel, using DCM/MeOH (5:1 *v*/*v*) as eluent, to afford **7** in 57% yield and presenting correct ESI-IT MS (+) data. m/z calculated for C_19_H_26_ClN_6_O: 389.19 a.m.u.; found: 389.73 ([M + H^+^]^+^).

#### 4.3.4. Synthesis of 2-Amino-*N*-{4-[(7-Methylquinolin-4-yl)amino]butyl}-3-sulfanylpropanamide, Cq-Cys (**8**)

The protected AA Boc-Cys(Trt)-OH (1.2 eq) was mixed with HBTU (1.2 eq) and DIPEA (2.4 eq) in DMF, and the solution was stirred in an ice-water bath, for 30 min. Then, compound **4** (1.0 eq) was added to the reaction mixture, which was left under stirring in the ice-water bath for another 3 h. The mixture was then diluted in ethyl acetate (20 mL) and extracted with an aqueous solution of 5% Na_2_CO_3_ (×3). The organic phase was dried with anhydrous Na_2_SO_4_ and, after filtration, ethyl acetate was removed by evaporation under reduced pressure. The resulting residue was then purified by column chromatography on silica gel, using DCM/MeOH (20:1 *v*/*v*) as eluent, to afford the protected Cq-Cys(Trt)-Boc intermediate. The Boc and Trt protecting groups were removed from this intermediate by acidolysis with TFA (95%), TIS (2.5%), and H_2_O (2.5%) for 1 h, in an ice-water bath. Crude product **8** was precipitated from the reaction mixture using cold *tert*-butyl methyl ether (TBME), the slurry centrifuged, the supernatant discarded, and the pellet re-suspended in fresh cold TBME. The procedure was repeated three more times, after which the solid pellet, corresponding to compound **8,** was dried in a desiccator (75% yield). Cq-Cys was finally analyzed by ESI-IT MS, and stored at ‒20 °C until further use. *m*/*z* calculated for C_16_H_21_ClN_4_OS: 352.11 a.m.u.; found: 353.47 ([M + H^+^]^+^).

#### 4.3.5. 2-Amino-*N*-{4-[(7-Methylquinolin-4-yl)amino]butyl}-3-(pyridin-2-yldisulfanyl)propaneimide, Cq-Cys(2-PDS) (**9**)

The thiol group in Cq-Cys (**8**) was activated upon reaction with 2-PDS. To this end, compound **8** (1.0 eq) and 2-PDS (2.0 eq) were dissolved in anhydrous methanol (4 mL) containing glacial acetic acid (0.3 eq). The mixture was stirred at RT for 18 h and the solvent was removed under reduced pressure to obtain the crude product. This was purified by flash chromatography on a two-step elution starting with DCM/MeOH (10:1 *v*/*v*) as eluent, followed by DCM/MeOH (1:1 *v*/*v*) as the second eluent. This afforded the target compound CQ-Cys(2-PDS) (**9**), in high purity and moderate yield (60%), and with correct mass spectral data. *m*/*z* calculated for C_21_H_24_ClN_5_OS_2_: 461.11 a.m.u.; found: 462.20 ([M + H]^+^).

### 4.4. Solid-Phase Synthesis

#### 4.4.1. General Procedures for Solid-Phase Peptide Synthesis

All target peptide sequences (Table 1) were produced as *C*-terminal amides by microwave-assisted solid-phase peptide synthesis. Generally, each peptide (*C*-terminal amide) was assembled by Fmoc/tBu SPPS methodologies, assisted with microwave energy, on a Rink amide MBHA resin that was preconditioned for 15 min in DMF and then transferred into the reaction vessel. The initial Fmoc deprotection step was carried out using 20% piperidine in DMF containing 0.1 M HOBt in two microwave irradiation pulses: 30 s at 24 W plus 3 min at 28 W, in both cases with temperature below 75 °C. The *C*-terminal AA was then coupled to the deprotected resin, using 5 eq of the Fmoc-protected AA (Fmoc-AA-OH) in DMF (0.2 M), 5 eq of 0.5 M HBTU/HOBt in DMF, and 10 eq of 2 M DIPEA in NMP; the coupling step was carried out for 5 min at 35 W microwave irradiation, with maximum temperature reaching 75 °C. The remaining AA were sequentially coupled in the *C*→*N* direction by means of similar deprotection and coupling cycles, except for the incorporation of Fmoc-Cys(Trt)-OH, which requires special attention during synthesis under microwave conditions in order to minimize the possibility of its racemization [47]. Hence, this amino acid was coupled in two steps, first with 2 min coupling without microwave irradiation (RT), then followed by 4 min coupling at 25 W, with maximum temperature reaching 50 °C. Following completion of the sequence, the *N*-terminal Fmoc-protecting group was removed with piperidine, as above, and the peptidyl-resin split in several portions to be either (i) cleaved to afford the unmodified (parent) CPP, or (ii) further modified (if needed) and coupled to compounds **6–9** (see below), then cleaved to afford the relevant drug-CPP conjugate. In either case, the target crude product was released from the resin with simultaneous removal of the AA side-chain protecting groups, by a 2-h acidolysis (RT) using a TFA-based cocktail containing either (i) TIS and deionized water as scavengers (TFA/TIS/H2O 95:2.5:2.5 *v*/*v*/*v*) for peptides with up to two arginine residues, or (ii) thioanisole, ethane-1,2-dithiol and anisole (90:5:3:2 *v*/*v*/*v*/*v*) for peptides with more than two arginines. Crude products were precipitated and washed with cold TBME, and purified by preparative HPLC. ESI-IT MS and analytical HPLC were used to check for the product’s molecular weights and purity. All peptides and respective conjugates were freeze-dried and stored at −20 °C until further use.

#### 4.4.2. Synthesis of Cq-C4-CPP Conjugates (**5a–5i**)

Each of the CPPs listed in Table 1 were coupled on-resin prior to cleavage to compound **6**, to afford peptide-drug conjugates **5a–5i**. To this end, **6** (5 eq) was pre-activated with PyBOP (5 eq) and DIPEA (10 eq) for 10 min (RT), and then added to each peptidyl-resin. The reaction was left overnight under stirring, then resin was washed three times with DMF and another three times with DCM. The final Cq-C4-CPP conjugates **5a–5i** were obtained after acidolytic cleavage, and purification by preparative HPLC, as above described.

#### 4.4.3. Synthesis of Cq-C10-TP10 Conjugate (**10**)

TP10 was assembled by microwave-assisted SPPS, as previously described, and its sequence further elongated upon coupling of an additional 6-aminohexanoyl (Ahx) residue at the *N*-terminus. To this end, Fmoc-Ahx-OH was coupled to the peptidyl-resin as described for standard AA. After removal of Fmoc and convenient washing of the resin, compound 6 was coupled to the resin-bound *N*-(6-aminohexanoyl)-TP10 peptide, as described for conjugates **5a–5i**. The target Cq-C10-TP10 conjugate (**10**) was obtained after cleavage and purification, as before.

#### 4.4.4. Synthesis of Cq-TR-TP10 Conjugate (**11**)

TP10 was assembled by microwave-assisted SPPS, as previously described, and its sequence further elongated upon coupling of an additional Fmoc-protected propargylglycine residue (Fmoc-Pra-OH) to the peptide’s *N*-terminus, and its Fmoc-protecting group next removed, by standard procedures in SPPS. Then, the resin-bound *N*-(propargylglycyl)-TP10 peptide was reacted with compound **7** (1.2 eq) in the presence of copper(I) bromide (1.2 eq), 2,6-lutidine (10 eq), sodium ascorbate (3 eq), and DIPEA (10 eq), solubilized in a mixture of ACN and DMF (1:2 *v*/*v*) [48]. The slurry was stirred overnight (RT), after which the resin was thoroughly washed with DCM (3×), MeOH (3×), ACN (3×), and DMF (3×). A 2-h acidolysis, using 95% TFA, 2.5% TIS, and 2.5% water, delivered crude product **11**, which was purified, analyzed, and stored as above.

#### 4.4.5. Synthesis of Cq-S-S-TP10 Conjugate (**12**)

TP10 was assembled by microwave-assisted SPPS as before, and its sequence further elongated upon coupling of its *N*-terminal AA to an additional Boc-Cys(NPys)-OH (5.0 eq), pre-activated with HBTU (5.0 eq) and DIPEA (10 eq), as usual. The peptidyl-resin was then cleaved as described above, to render the peptide intermediate H-Cys(NPys)-TP10 (**12**). This intermediate (1.0 eq) was next mixed with compound **8** (1.2 eq) in 1 M aqueous acetic acid, and the thiol-disulfide exchange reaction allowed to proceed for 36 h under stirring (RT). Reaction progress was monitored by HPLC and LC-DAD/ESI-IT MS. The target Cq-S-S-TP10 conjugate (**12**) was purified, analyzed and stored as described for the other conjugates.

#### 4.4.6. Synthesis of TP10-C4-Cq Conjugate (**13**)

Peptide TP10 was assembled by microwave-assisted SPPS as previously described, but using a Fmoc-Lys(Mtt)-OH residue on position **19** of the peptide sequence (bold in AGYLLGKINLKALAALAK**K**IL), enabling selective side-chain deprotection of this particular residue via mild acidolytic conditions without peptide cleavage from the resin [49,50]. Once the peptide sequence was fully assembled, and prior to removal of the *N*-terminal Fmoc protecting group, the peptidyl-resin was treated with 1% TFA in DCM, for selective removal of the Mtt protecting group; this procedure was repeated until the yellow color associated with Mtt release was no longer observed. Then, after convenient washing of the peptidyl-resin, compound **6** was coupled to the lysine side chain ε-amine, by in situ activation with PyBOP (5.0 eq) and DIPEA (10 eq), in DMF. This reaction was left overnight under stirring (RT), after which the resin was filtered and washed with DCM (3×) and DMF (3×). The crude conjugate **13** was obtained by acidolytic cleavage, then purified, analyzed, and stored, as previously described for the other conjugates.

#### 4.4.7. Synthesis of TP10-S-S-Cq Conjugate (**14**)

A TP10 derivative bearing an additional *C*-terminal cysteine residue, or briefly, peptide TP10-Cys (AGYLLGKINLKALAALAKKIL**C**), was assembled by standard microwave-assisted SPPS procedures, and cleaved by acidolysis, as done for all other peptides. The peptide (1.0 eq) was then mixed with compound **9** (1.2 eq) in 1 M aqueous acetic acid, and the thiol-exchange reaction allowed to proceed for 48 h, at RT. This reaction was monitored by HPLC, with a gradient elution of 1–100% ACN in 0.05% aqueous TFA, and by LC-DAD/ESI-IT MS. The target peptide conjugate, TP10-S-S-Cq (**14**) was purified, analyzed, and stored as described for the other conjugates.

#### 4.4.8. Synthesis of Fluorescently-Labeled TP10-K(CF) (**15**) and TAT-K(CF) Peptides

TP10 and TAT derivatives bearing an additional *C*-terminal lysine, i.e., TP10-Lys (AGYLLGKINLKALAALAKKILK) and TAT-Lys (RKKRRQRRRPPQK) were synthesized via standard microwave-assisted SPPS, using Fmoc-Lys(Mtt)-OH (5.0 eq) as the *C*-terminal residue. Once each peptide sequence was fully assembled, and prior to removal of the *N*-terminal Fmoc protecting group, the peptidyl-resin was treated with 1% TFA in DCM, for selective removal of the Mtt protecting group; this procedure was repeated until the yellow color associated with Mtt release was no longer observed. Then, after convenient washing of the peptidyl-resin, 5(6)-carboxyfluorescein (CF) was coupled to the lysine side chain ε-amine, by in situ activation with PyBOP (5.0 eq) and DIPEA (10 eq), in DMF. This reaction proceeded for 2 h under stirring (RT), after which the resin was filtered and washed with DCM (3×) and DMF (3×). The Fmoc-protected peptidyl-resin bearing the TP10-based sequence was split into two equal portions, one of which was treated with piperidine for removal of the *N*-terminal Fmoc-protecting group, and next cleaved by acidolysis to release the fluorescently-labeled peptide **15**, which was purified, analyzed and stored as before. The other portion was stored for subsequent on-resin coupling to compound **6** (see below). Likewise, the Fmoc-protected peptidyl-resin bearing the TAT-based sequence was stored for subsequent coupling to compound **6** (see below).

#### 4.4.9. Synthesis of Fluorescently Labeled Cq-C4-TP10-K(CF) (**16**) and Cq-C4-TAT-K(CF) (**17**) Conjugates

Fmoc-protected peptidyl resins bearing the fluorescently labeled peptides TP10 and TAT, prepared as described above, were treated with 20% piperidine in DMF, to remove the *N*-terminal Fmoc protecting group. Next, compound 6 was coupled to the peptidyl-resin, upon activation with PyBOP (5.0 eq) and DIPEA (10.0 eq), in DMF. This reaction was kept under stirring overnight (RT), after which the resin was filtered and washed with DCM (3×) and DMF (3×). The crude fluorescently-labeled conjugates **16** and **17** were obtained after a 2 h cleavage (RT) of the peptidyl-resins with a solution of TFA (95%), TIS (2.5%), and water (2.5%). The conjugates were purified, analyzed, and stored as above.

### 4.5. In Vitro Assessment of Antimalarial Activity

#### 4.5.1. Activity of Conjugates **3a** and **5a-i** against *P. Falciparum* W2

Synchronized ring-stage *P. falciparum* parasites (W2 strain) were incubated with test compounds for 48 h in Roswell Park Memorial Institute (RPMI) 1640 medium containing 10% human serum or 0.5% Albumax serum substitute. Parasites were then fixed with 1% formaldehyde in phosphate-buffered saline (PBS), pH 7.4, for 48 h at RT and labeled with YOYO-1 (1 nM; molecular probes) in 0.1% Triton X-100 in PBS. Parasitemias were determined by flow cytometry, from dot plots (forward scatter versus fluorescence). For 50% inhibitory concentration (IC_50_) determinations, dose-response studies were performed, with the values normalized to percent control activity, and IC_50_s were calculated using GraphPad Prism, with data fitted by nonlinear regression.

#### 4.5.2. Activity of Conjugates **3a**, **5a**, **14′** and **11–15** against *P. Falciparum* 3D7

Synchronized ring-stage *P. falciparum* (3% hematocrit, 1% parasitemia) were incubated in a microtiter plate for 48 h in the presence of two-fold serial dilutions of TP10 and derived conjugates 11–15, in the 10–10000 nM concentration range. Cultures incubated with three-fold serial dilutions of chloroquine bisphosphate, and also of its analogue 4, in the 0.1–8000 nM concentration range, were included for comparison. Culture without test compounds was also added to the microtiter plate, as negative control. After incubation, 100 μL of a solution of SYBR Green (Thermo Fisher; SG; 0.001% *v*/*v* in PBS) was added to each well and plates incubated for another 60 min under standard culture conditions. Plates were then centrifuged at 2500 rpm for 2 min, the supernatant was discarded, and cells were re-suspended in 100 μL of PBS. Fluorescence was collected in a multi-mode microplate reader (Triad, Dynex Technologies) with excitation at 485 nm and emission at 535 nm [37]. Fluorescence values were normalized (to take into account the normal growth of the parasite) and then plotted against logC. IC_50_ were determined using GraphPad Prism 6.

### 4.6. In Vitro Assessment of Hemolytic Activity

PiRBC and hRBC were plated in micro-titer plates and cultured for 1 h in the presence of four-fold serial dilutions of Cq, TP10 and their conjugates (40–10000 nM). For positive and negative controls, 1% Triton X-100 and PBS were added, respectively. After this period, the plates were centrifuged and supernatants transferred to another plate. Supernatant absorbance at 450 nm was assessed in a multi-mode microplate reader (Triad, Dynex Technologies, Chantilly, VA, USA) and values were treated using the following equation (1):

(1) $$\%\;\mathrm{Hemolysis}=\frac{Abs-Abs\left(negative\;control\right)}{Abs\left(positive\;control\right)-Abs\left(negative\;control\right)}$$

### 4.7. Fluorescence Microscopy and Flow Cytometry Studies

#### 4.7.1. Fluorescence Microscopy Assays

PiRBC suspensions (3% hematocrit, 3% parasitemia) were incubated for 1 h with CF-labeled conjugates **15** or **16** (to a final concentration of 8 µM), at 37 °C. After incubation, cells were washed twice with culture medium and finally re-suspended in a solution of 2 µg/mL of Hoescht 33342 in culture medium, in order to stain the PiRBC nuclei. Cells were then diluted to 0.1% hematocrit in RPMI 1640, and finally deposited onto a Lab-TekTM chambered coverglass slide. Microscopic analysis was performed with an Olympus IX51 inverted system microscope, equipped with an IX2-SFR X-Y stage, a U-TVIX-2 camera, and a fluorescence mirror unit cassette for UV/blue/green excitation and detection of the corresponding blue/green/red emission ranges.

#### 4.7.2. Flow Cytometry

Flow cytometry was used to determine the targeting efficiency of fluorescently labeled TP10 (15) and conjugates **16** and **17** towards an unsynchronized *P. falciparum* 3D7 culture. To this end, parasitemia was adjusted to approximately 3% and the culture was incubated for 1 h at 37 °C in the presence of the labeled compounds (8 μM). After incubation, the suspensions were washed twice with RPMI complete medium and finally re-suspended in a solution of 2 µg/mL of Hoechst 33342 in RPMI 1640, in order to stain PiRBC nuclei. Samples were then diluted in PBS to a final concentration of 10^7^ cells/mL, prior to flow cytometry analysis. Forward- and side-scatter areas (FSC-A, SSC-A) in a linear scale were used to gate the RBC population, and PiRBC stained with Hoescht 33342 were detected by excitation through a 350 nm laser and emission collection with a 450/50 nm bandpass filter in a logarithmic scale. RBC targeted by CF-labeled test compounds were detected by excitation through a 488 nm laser and emission collection with a 525/50 nm bandpass filter in a logarithmic scale.

## 5. Conclusions

The disclosure of CPP as carriers for a wide range of payloads has been an undeniable scientific breakthrough, paving the way towards targeted intracellular delivery of many different types of cargo, from small anticancer drugs to large proteins and nucleic acids [51,52,53,54]. To the best of our knowledge, markedly increased hemolysis upon conjugation of CPP to drugs has never been reported before, which highlights the relevance of assessing hemolytic activity of any newly developed CPP-cargo conjugate. The heteroaromatic structure of the antimalarial drugs used is apparently relevant for the observed hemolytic effects, as these were not described for previously reported fosmidomycin-CPP conjugates displaying antimalarial activity [16]. Ongoing biophysical studies will hopefully clarify the molecular mechanisms that are responsible for the observations herein reported, towards future strategies that may overcome this hurdle.

## Figures and Tables

**Table 1:** Selected CPP, their molecular weights (MW), and MW of respective conjugates **5a-5i**.

CPP	Amino Acid Sequence (Respective MW/Da)	Cq-C4-CPP Conjugate (Respective MW/Da)
TP10	AGYLLGKINLKALAALAKKIL (2182)	**5a** (2512)
Transportan	GWTLNSAGYLLGKINLKALAALAKKIL (2840)	**5b** (3170)
DPT-sh1	VKKKKIKREIKI (1510)	**5c** (1841)
DPT-sh2	RQKRLIRQKRLIRQKRLI (2402)	**5d** (2732)
IDR-1018	VRLIVAVRIWRR (1535)	**5e** (1867)
TAT	GRKKRRQRRRPPQ (1718)	**5f** (2049)
PasTAT	FFLIPKGGRKKRRQRRRPPQ (2521)	**5g** (2852)
R9	RRRRRRRRR (1423)	**5h** (1753)
Penetratin	RQIKIWFQNRRMKWKK (2246)	**5i** (2576)

**Table 2:** Activity of unmodified CPP and respective conjugates **5a**-**5i** against *P. falciparum* W2.

CPP	IC_50_/µM	SD (*n* = 3)	Cq-C4-CPP	IC_50_/µM	SD (*n* = 3)
TP10	5.5	0.2	Cq-C4-TP10, **5a**	1.5	0.1
Transportan	3.1	0.8	Cq-C4-Transportan, **5b**	5.2	0.2
DPT-sh1	>10	-	Cq-C4-DPT-sh1, **5c**	>10	-
DPT-sh2	>10	-	Cq-C4-DPT-sh2, **5d**	>10	-
IDR-1018	>10	-	Cq-C4-IDR-1018, **5e**	>10	-
TAT	>10	-	Cq-C4-TAT, **5f**	>10	-
PasTAT	>10	-	Cq-C4-PasTAT, **5g**	8.5	0.2
R9	>10	-	Cq-C4-R9, **5h**	>10	-
Penetratin	>10	-	Cq-C4-Penetratin, **5i**	>10	-
CQ (1)	0.699 ^a^				

^a^ Value taken from [22].

**Table 3:** Antiplasmodial (*P. falciparum* 3D7) and hemolytic activity of conjugates **3a**, **5a**, **10-14**, **14′**.

Test Compound	MW/Da	IC_50_ ± SD (*n* = 3)/µM	% hemolysis at 10 µM ± SD (*n* = 3)	
			hRBC	PiRBC
PQ-C4-TP10, **3a**	2525	- ^a^	89 ± 8	49 ± 7
Cq-C4-TP10, **5a**	2514	0.8 ± 0.1	23 ± 1	6.5 ± 0.4
Cq-C10-TP10, **10**	2626	0.8 ± 0.3	38 ± 2	19 ± 1
Cq-TR-TP10, **11**	2665	1.2 ± 0.2	23 ± 1	10.7 ± 0.1
Cq-S-S-TP10, **12**	2636	1.0 ± 0.3	8.5 ± 0.5	2.0 ± 0.3
TP10-C4-Cq, **13**	2514	- ^a^	50 ± 2	41 ± 2
TP10-S-S-Cq, **14**	2636	2.3 ± 0.4	18.2 ± 0.8	7 ± 1
TP10-S-S-PQ, **14′**	2647	- ^a^	99.7 ± 0.4	72 ± 9
TP10	2182	1.9 ± 0.5	3.7 ± 0.1	3.4 ± 0.2
CQ, **1**	319.9	0.021^b^	- ^c^	- ^c^
Cq, **4**	249.7	0.07 ± 0.02	- ^c^	- ^c^
Cq + TP10 1:1	n.a.	0.024^d^	4.4 ± 0.6	1.8 ± 0.5

^a^ Consistent growth inhibition data could not be obtained; ^b^ taken from reference [2]; ^c^ hemolysis not detected; ^d^ SD (*n* = 3) were too high, this should be considered an orientative, not an accurate value; n.a., not applicable.

## Supplementary Materials

The following are available online at https://www.mdpi.com/1420-3049/24/24/4559/s1, Figures S1 to S55: ESI-IT MS spectra and chromatograms obtained for the synthesized compounds.
