# Supplementary material for: Coupling the Antimalarial Cell Penetrating Peptide TP10 to Classical Antimalarial Drugs Primaquine and Chloroquine Produces Strongly Hemolytic Conjugates
Source: Molecules. 2019 Dec 12;24(24):4559. doi: 10.3390/molecules24244559 (PMC6943437; doi:10.3390/molecules24244559)
Supplement: Supplementary file 1 [file molecules-24-04559-s001.pdf]

Supporting information

Coupling the antimalarial cell penetrating peptide TP10 to classical antimalarial drugs primaquine and chloroquine produces strongly hemolytic conjugates

Luísa Aguiar [1], Arnau Biosca [2,3], Elena Lantero [2,3], Jiri Gut [4], Nuno Vale [5,6,7], Philip J. Rosenthal [4], Fátima Nogueira [8], David Andreu [9], Xavier Fernàndez-Busquets [2,3,10] and Paula Gomes [1,*]

1 LAQV-REQUIMTE, Departamento de Química e Bioquímica, Faculdade de Ciências, Universidade do Porto, 4169-007 Porto, Portugal; luisa.aguiarts@gmail.com
2 Barcelona Institute for Global Health (ISGlobal, Hospital Clínic-Universitat de Barcelona), Rosselló 149-153, 08036 Barcelona, Spain; abiosca@ibecbarcelona.eu (A.B.); elantero@ibecbarcelona.eu (E.L.); xfernandez_busquets@ub.edu (X.F.-B.)
3 Nanomalaria Group, Institute for Bioengineering of Catalonia (IBEC), The Barcelona Institute of Science and Technology, Baldiri Reixac 10-12, 08028 Barcelona, Spain
4 School of Medicine, University of California at San Francisco, 1001 Potrero Avenue, San Francisco, San Francisco, CA 94110, USA; jiri.gut@ucsf.edu (J.G.); Philip.Rosenthal@ucsf.edu (P.J.R.)
5 Departamento de Farmacologia, Departamento de Ciências do Medicamento, Faculdade de Farmácia da Universidade do Porto, Rua de Jorge Viterbo Ferreira 228, 4050-313 Porto, Portugal; nuno.vale@ff.up.pt
6 IPATIMUP – Instituto de Patologia e Imunologia Molecular da Universidade do Porto, Rua Júlio Amaral de Carvalho 45, 4200-135 Porto, Portugal
7 i3S, Instituto de Investigação e Inovação em Saúde, Rua Alfredo Allen 208, 4200-135 Porto, Portugal
8 Global Health and Tropical Medicine, Instituto de Higiene e Medicina Tropical, Universidade Nova de Lisboa, 1349-008 Lisbon, Portugal; FNogueira@ihmt.unl.pt
9 Department of Experimental and Health Sciences, Pompeu Fabra University, Barcelona Biomedical Research Park, Dr. Aiguader 88, 08003 Barcelona, Spain; david.andreu@upf.edu
10 Nanoscience and Nanotechnology Institute (IN2UB), University of Barcelona, Martí i Franquès 1, 08028 Barcelona, Spain

* Correspondence: pgomes@fc.up.pt

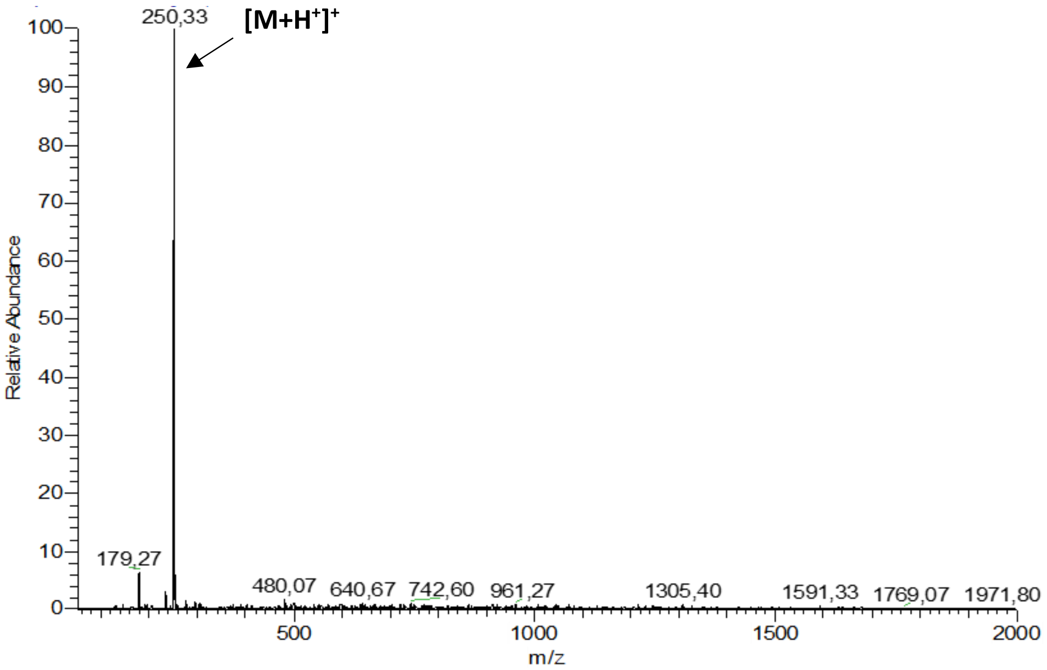

Figure S1 – ESI-IT MS spectrum (positive mode) of Cq (**4**)

(observed as the quasi-molecular ion)

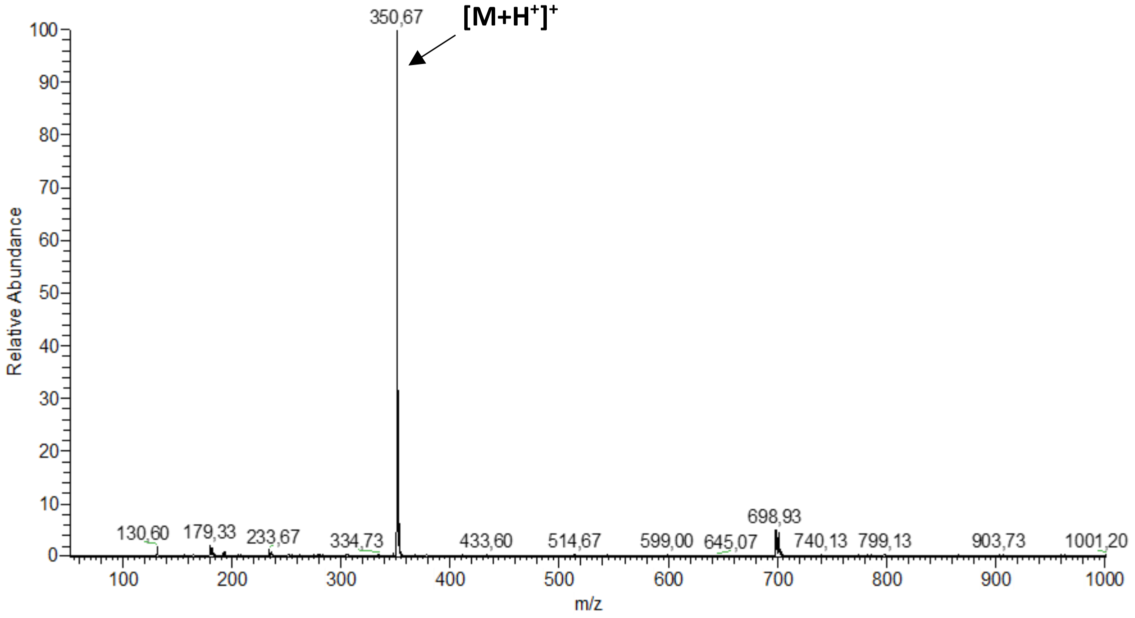

Figure S2 – ESI-IT MS spectrum (positive mode) of Cq-C4 (**6**)

(observed as the quasi-molecular ion)

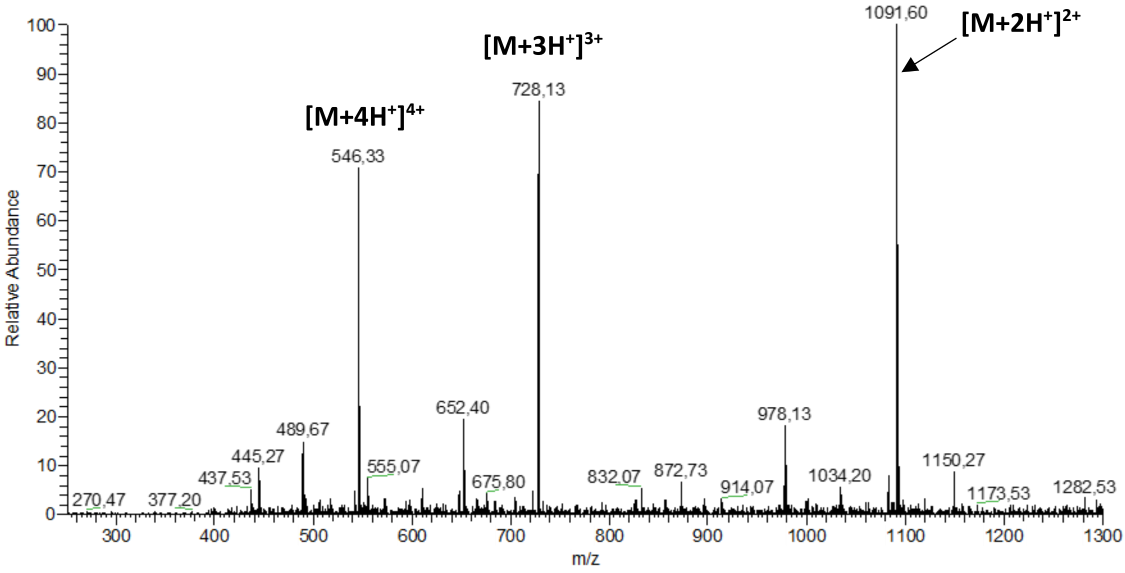

Figure S3 - ESI-IT MS spectrum (positive mode) of peptide TP10

(observed as the di-, tri-, and tetra-protonated ions)

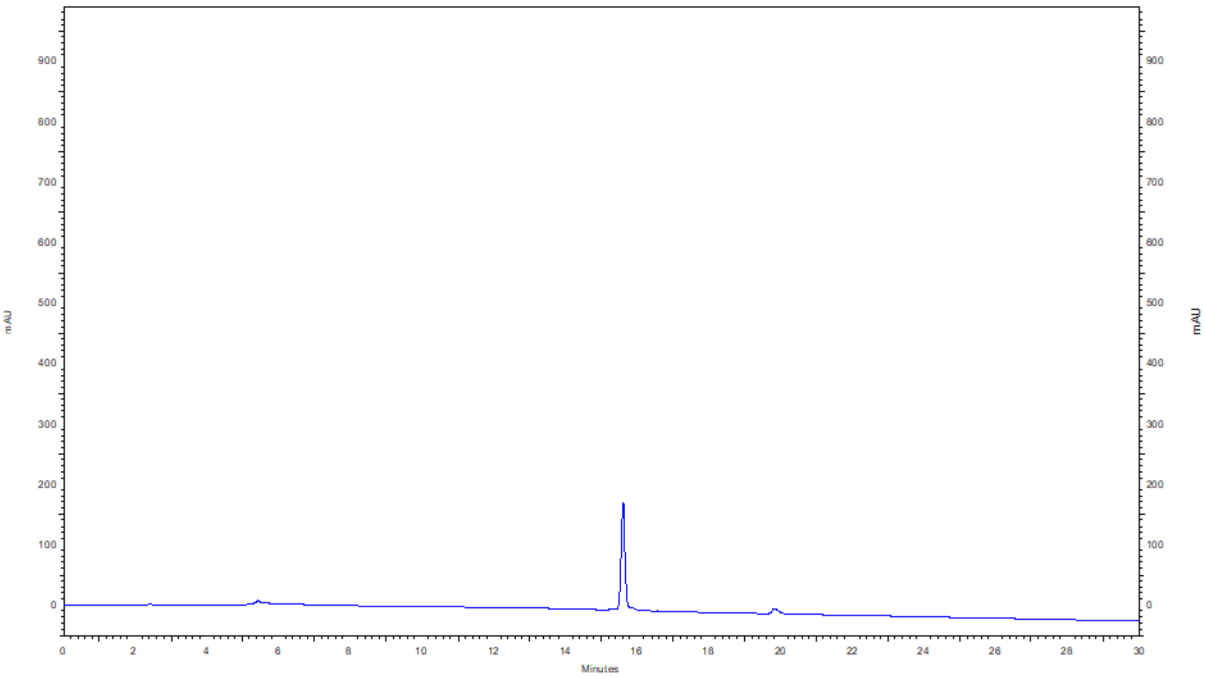

Figure S4 – Chromatogram obtained for TP10, with a gradient elution of 0-100% ACN in water (0.05% TFA) in a reverse phase C18 column Purospher star RP-C18 of 125 × 4.0 mm with 5 μm pore size, for 30 min and at a flow rate of 1 mL/min, with detection at λ=220 nm.

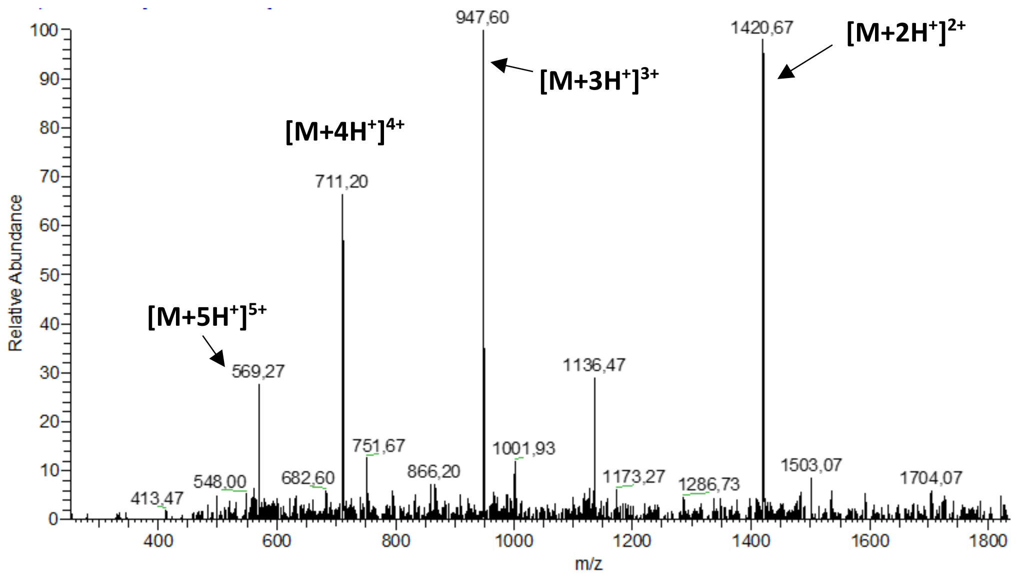

Figure S5 - ESI-IT MS spectrum (positive mode) of peptide Transportan (observed as the di-, tri-, tetra-, and penta-protonated ions)

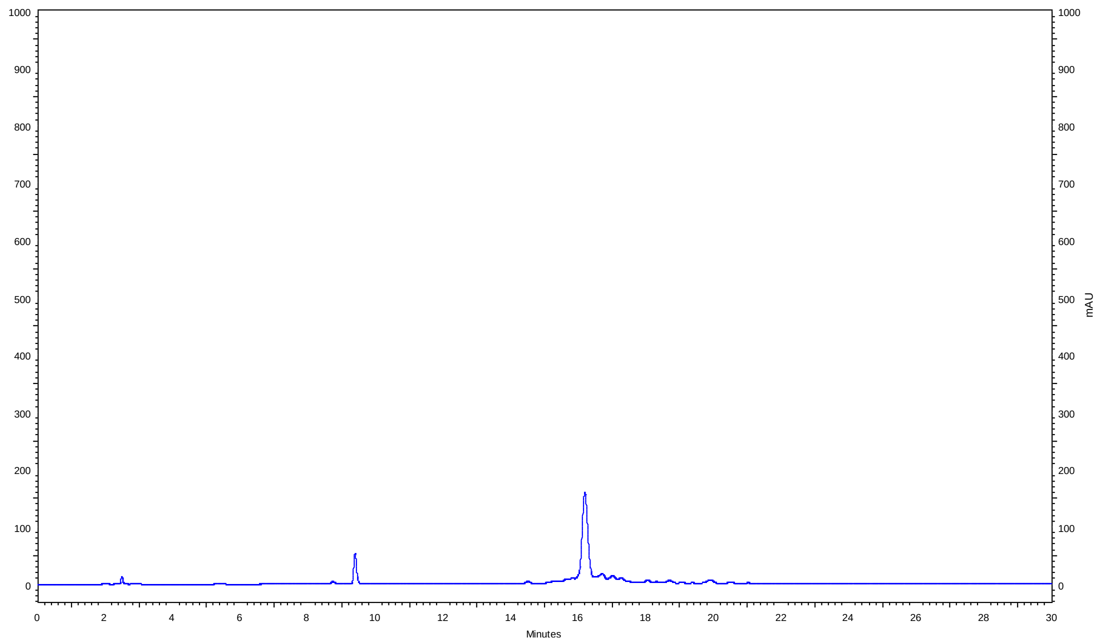

Figure S6 – Chromatogram obtained for Transportan, with a gradient elution of 0-100% ACN in water (0.05% TFA) in a reverse phase C18 column Purospher star RP-C18 of 125 × 4.0 mm with 5 µm pore size, for 30 min and at a flow rate of 1 mL/min, with detection at λ=220 nm.

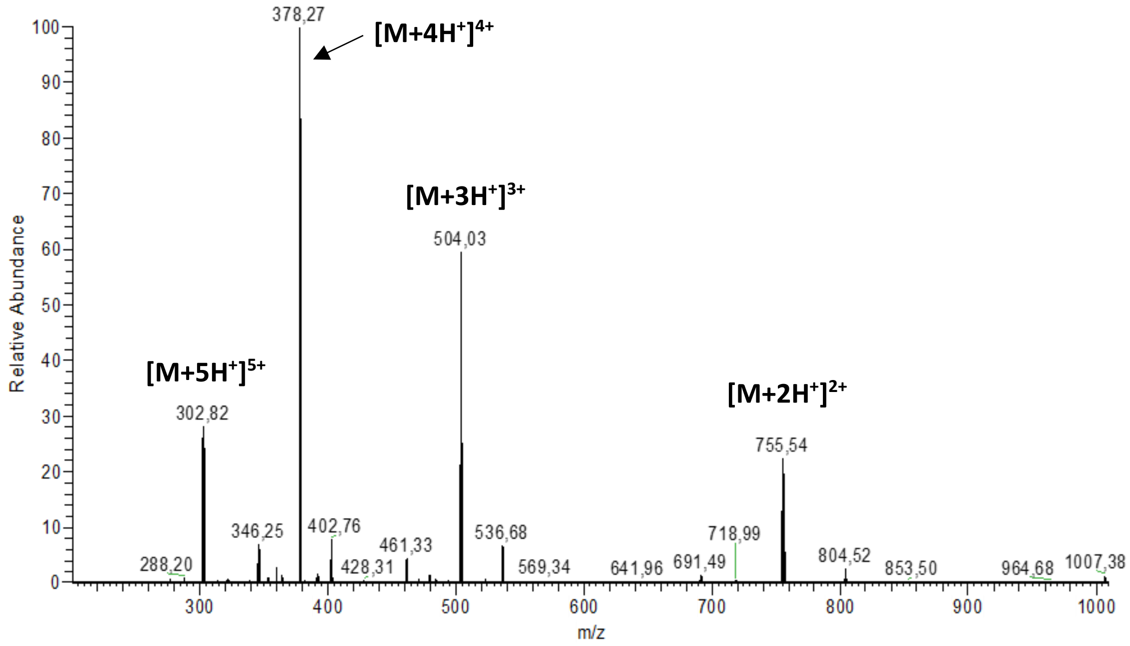

Figure S7 - ESI-IT MS spectrum (positive mode) of peptide DPT-sh1

(observed as the di-, tri-, tetra-, and penta-protonated ions)

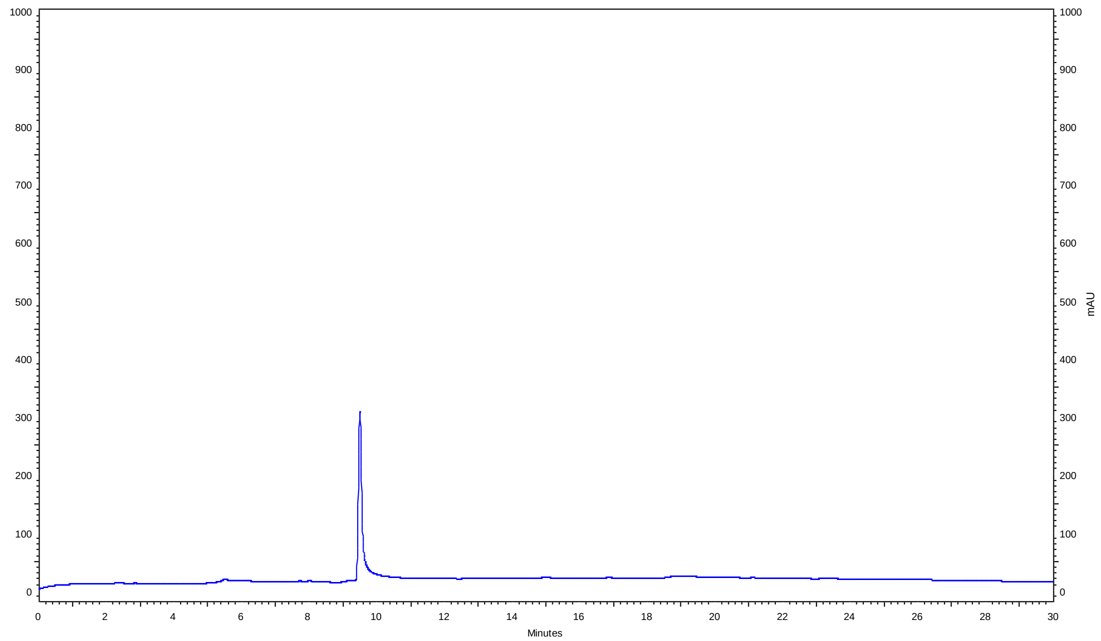

Figure S8 – Chromatogram obtained for DPT-sh1, with a gradient elution of 0-100% ACN in water (0.05% TFA) in a RP-18E (5 µm), for 30 min and a flow rate of 1 ml/min, with detection at λ=220 nm.

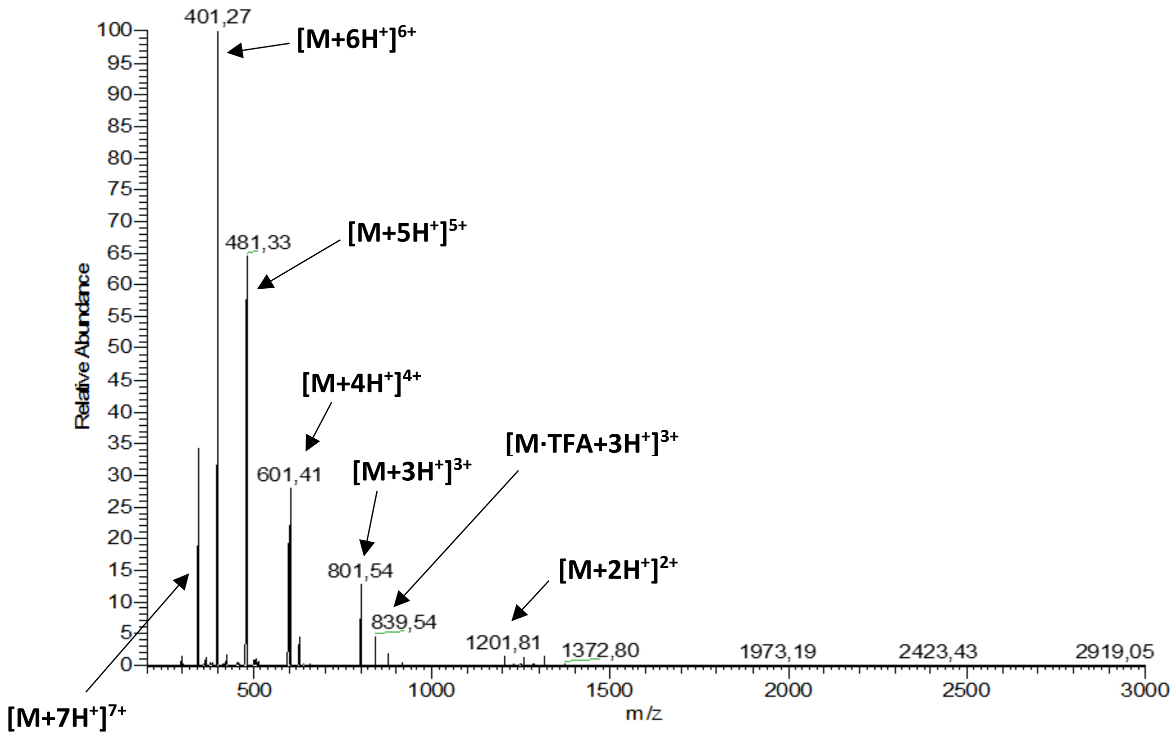

Figure S9 - ESI-IT MS spectrum (positive mode) of peptide DPT-sh2 (observed as the di-, tri-, tetra-, penta-, hexa-, and hepta-protonated ions; TFA adducts at different protonation states also observed)

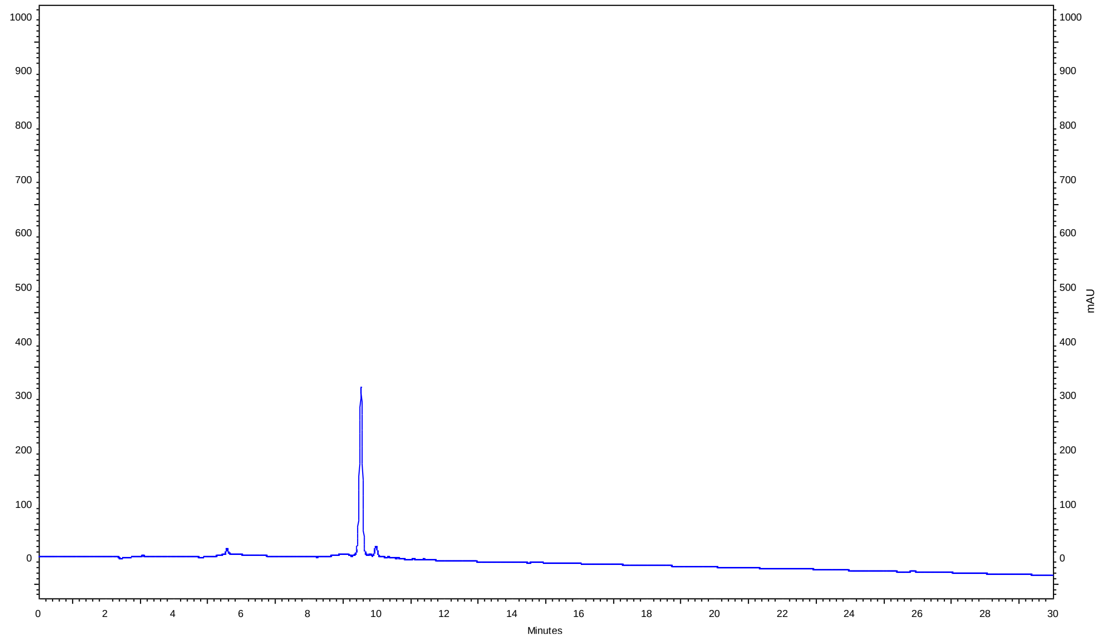

Figure S10 – Chromatogram obtained for DPT-sh2, with a gradient elution of 0-100% ACN in water (0.05% TFA) in a RP-18E (5 μm), for 30 min and a flow rate of 1 ml/min, with detection at λ=220 nm.

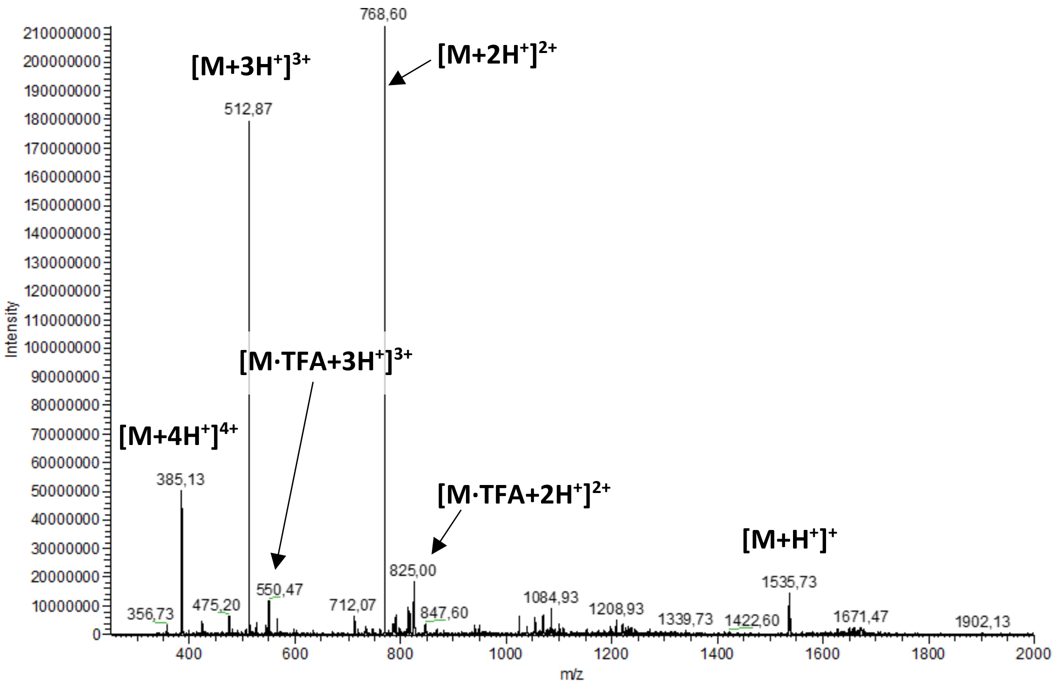

Figure S11 - ESI-IT MS spectrum (positive mode) of peptide IDR-1018

(observed as the quasi-molecular ion, and also as the di-, tri-, and tetra-protonated ions; TFA adducts at different protonation states also observed)

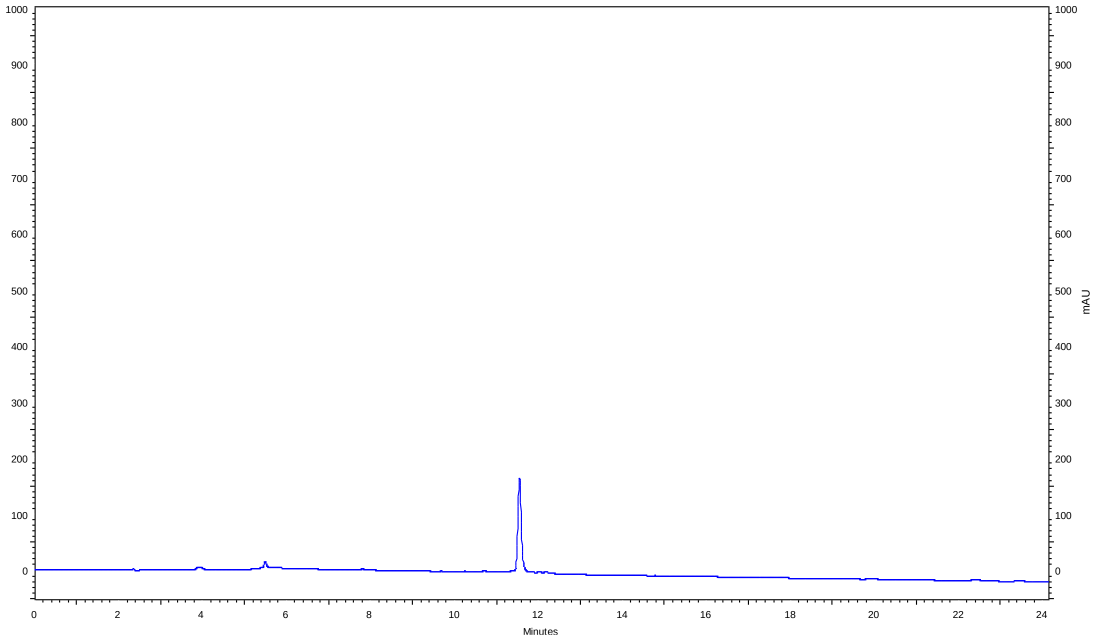

Figure S12 – Chromatogram obtained for IDR-1018, with a gradient elution of 0-100% ACN in water (0,05% TFA) in a RP-18E (5 μm), for 30 min and a flow rate of 1 ml/min, with detection at λ=220 nm.

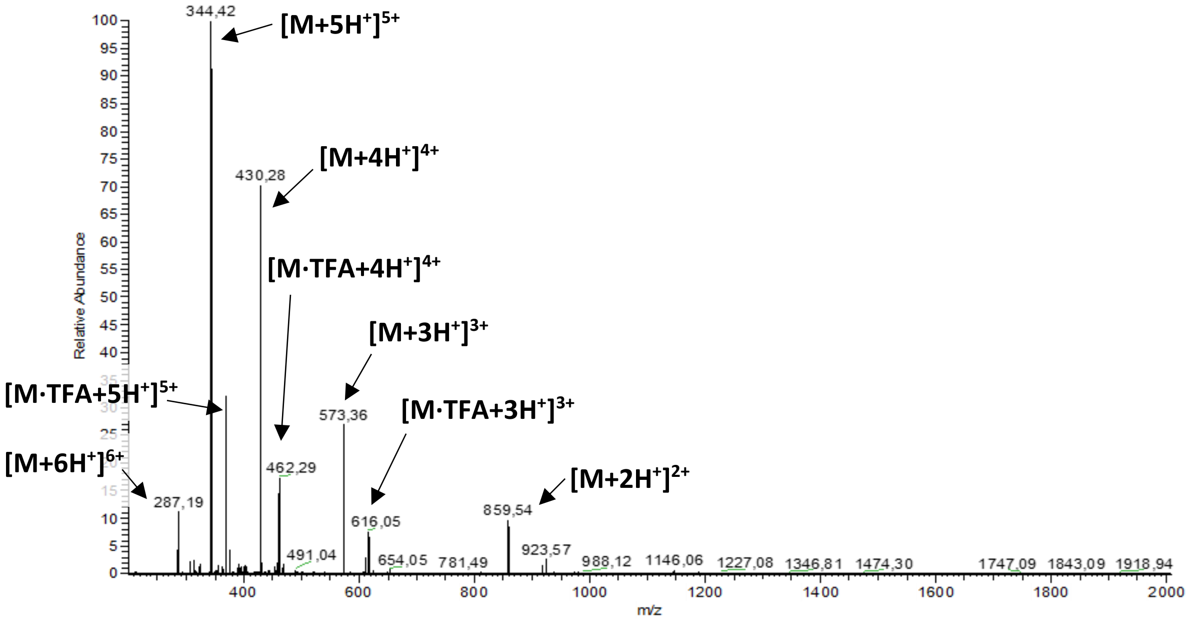

Figure S13 - ESI-IT MS spectrum (positive mode) of peptide TAT (observed as the di-, tri-, tetra-, penta-, and hexa-protonated ions; TFA adducts at different protonation states also observed)

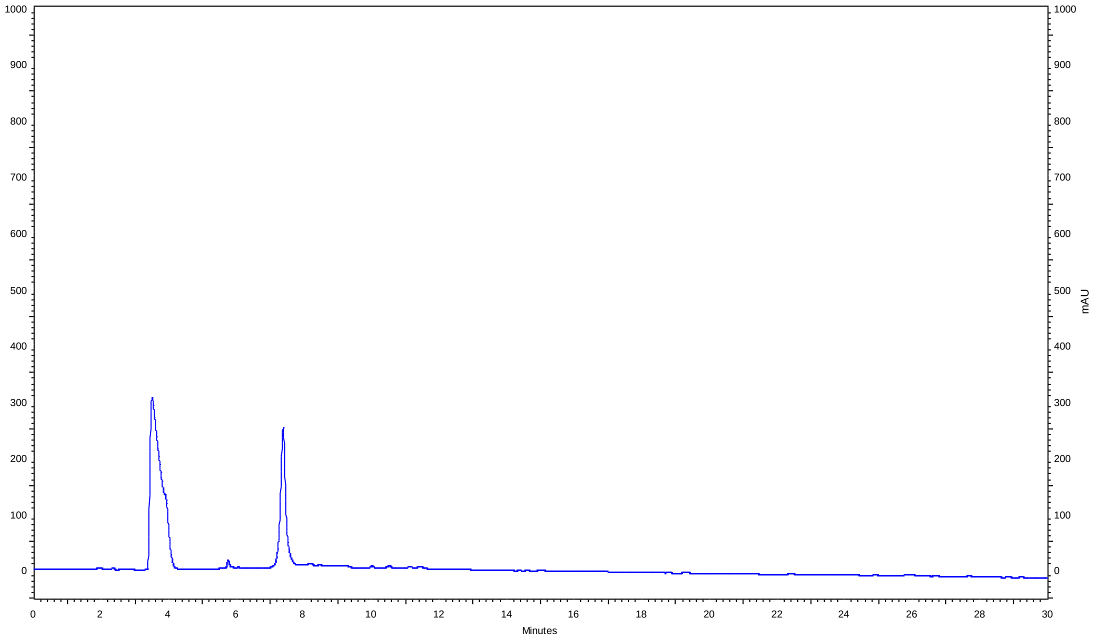

Figure S14 – Chromatogram obtained for TAT, with a gradient elution of 0-100% ACN in water (0,05% TFA) in a RP-18E (5 μm), for 30 min and a flow rate of 1 ml/min, with detection at λ=220 nm. The peak at RT ≈ 3-4 min is due to acetic acid, added to the samples for peptide solubilization.

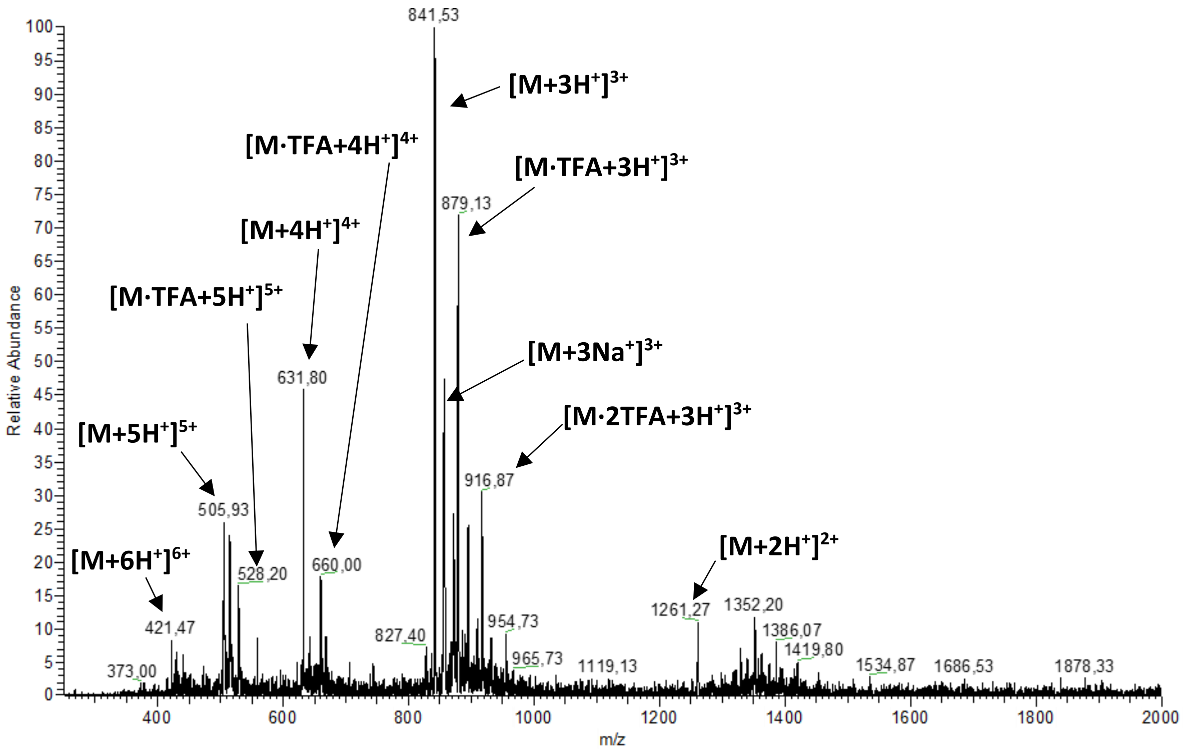

Figure S15 - ESI-IT MS spectrum (positive mode) of peptide PasTAT (observed as the di-, tri-, tetra-, penta-, and hexa-protonated ions; sodium and TFA adducts at different protonation states also observed)

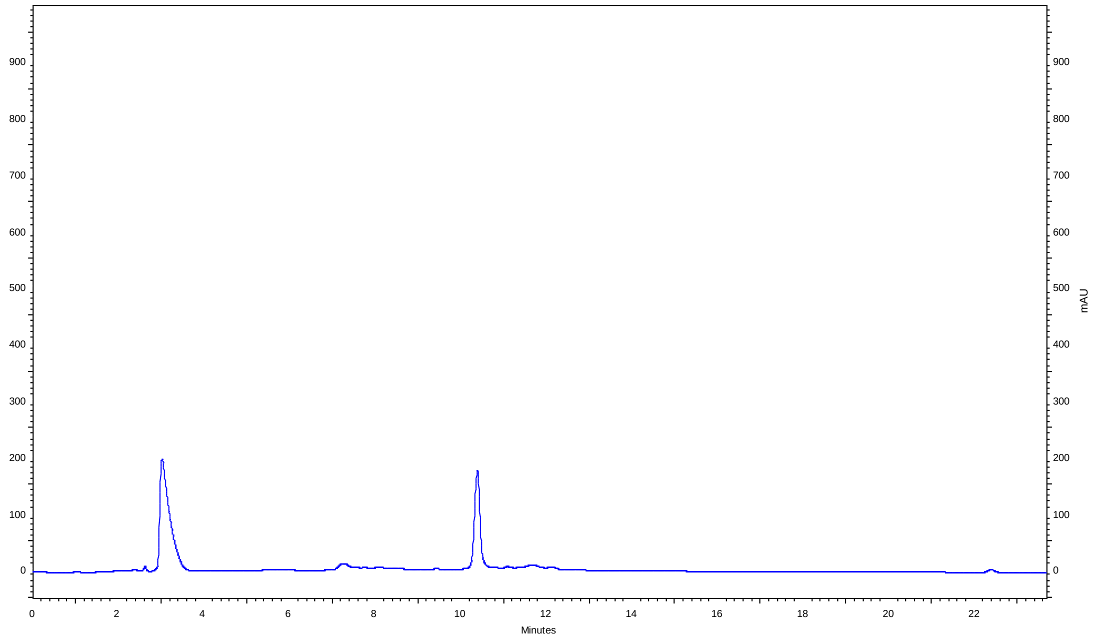

Figure S16 – Chromatogram obtained for PasTAT, with a gradient elution of 0-100% ACN in water (0,05% TFA) in a RP-18E (5 µm), for 30 min and a flow rate of 1 ml/min, with detection at λ=220 nm. The peak at RT ≈ 3 min is due to acetic acid, added to the samples for peptide solubilization.

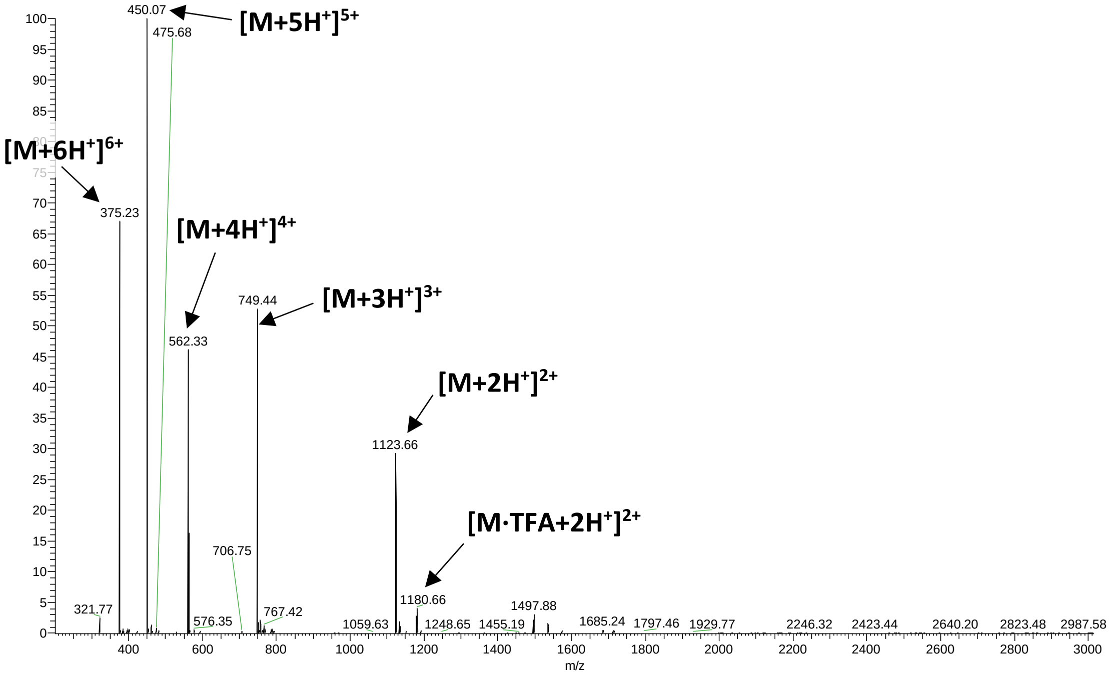

Figure S17 - ESI-IT MS spectrum (positive mode) of peptide Penetratin (observed as the di-, tri-, tetra-, penta-, and hexa-protonated ions; TFA adducts at different protonation states also observed)

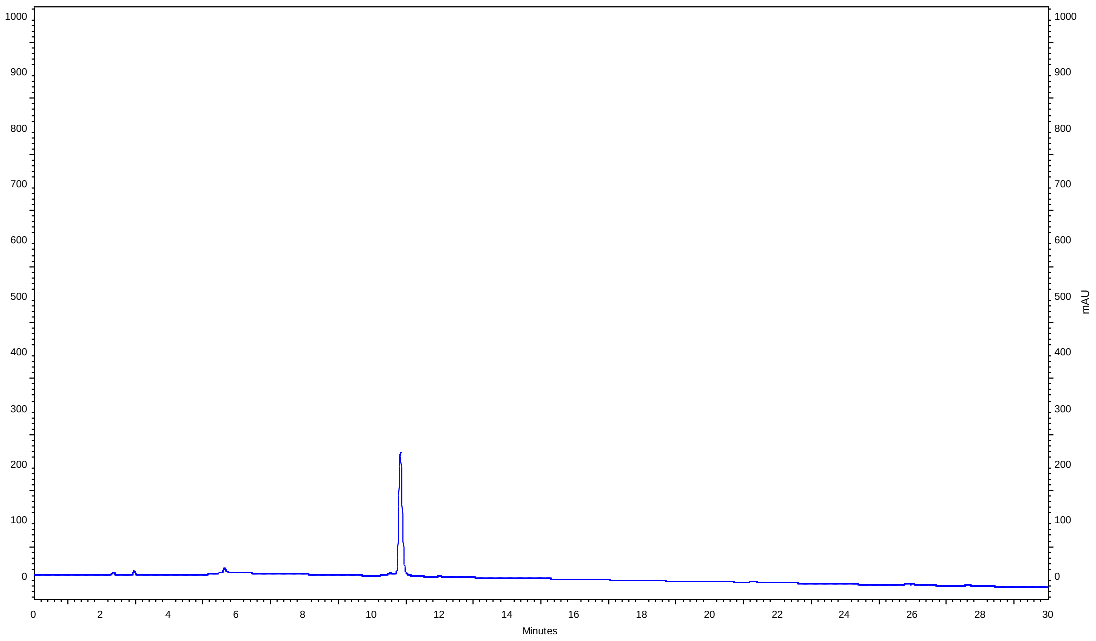

Figure S18 - Chromatogram obtained for Penetratin, with a gradient elution of 0-100% ACN in water (0,05% TFA) in a RP-18E (5 μm), for 30 min and a flow rate of 1 ml/min, with detection at λ=220 nm.

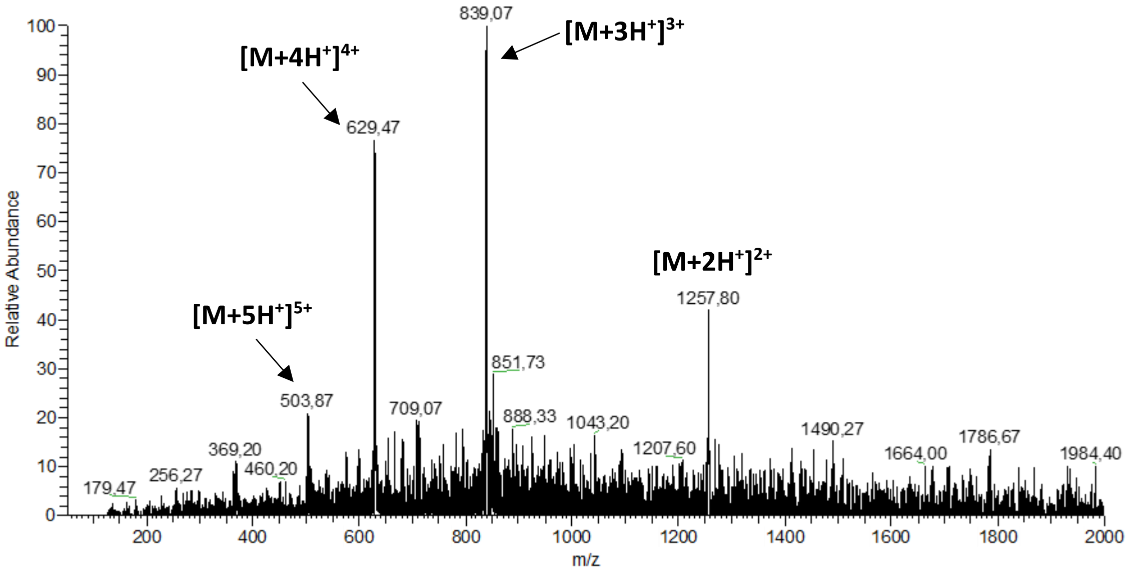

Figure S19 - ESI-IT MS spectrum (positive mode) of conjugate Cq-C4-TP10 (**5a**) (observed as the di-, tri-, tetra-, and penta-protonated ions)

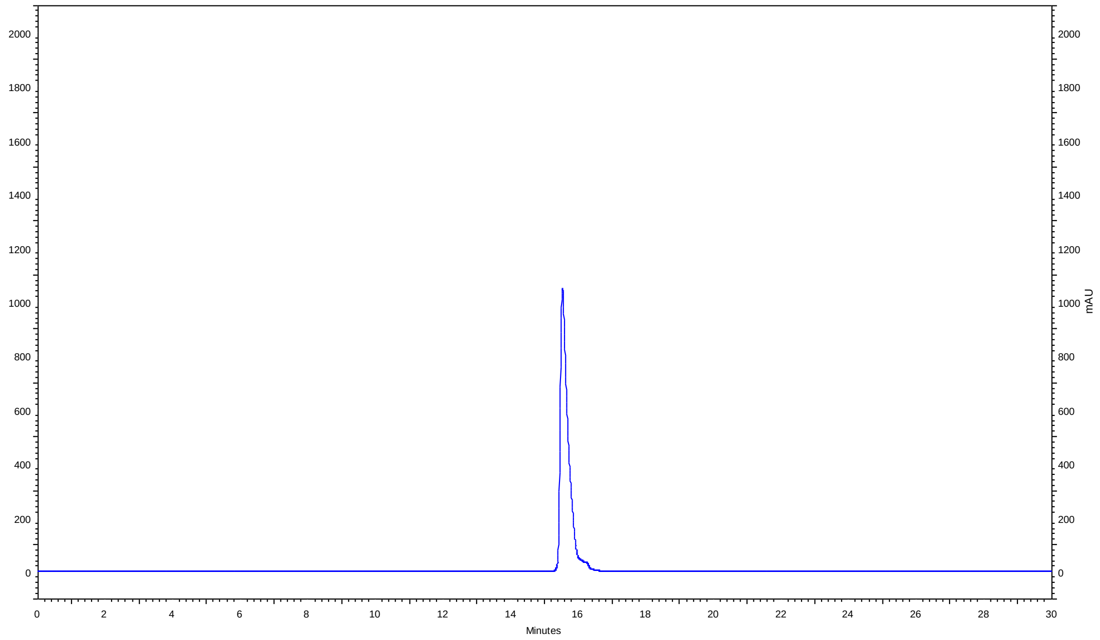

Figure S20 – Chromatogram obtained for Cq-C4-TP10 (**5a**), with a gradient elution of 0-100% ACN in water (0,05% TFA) in a RP-18E (5 μm), for 30 min and a flow rate of 1 ml/min, with detection at λ=220 nm.

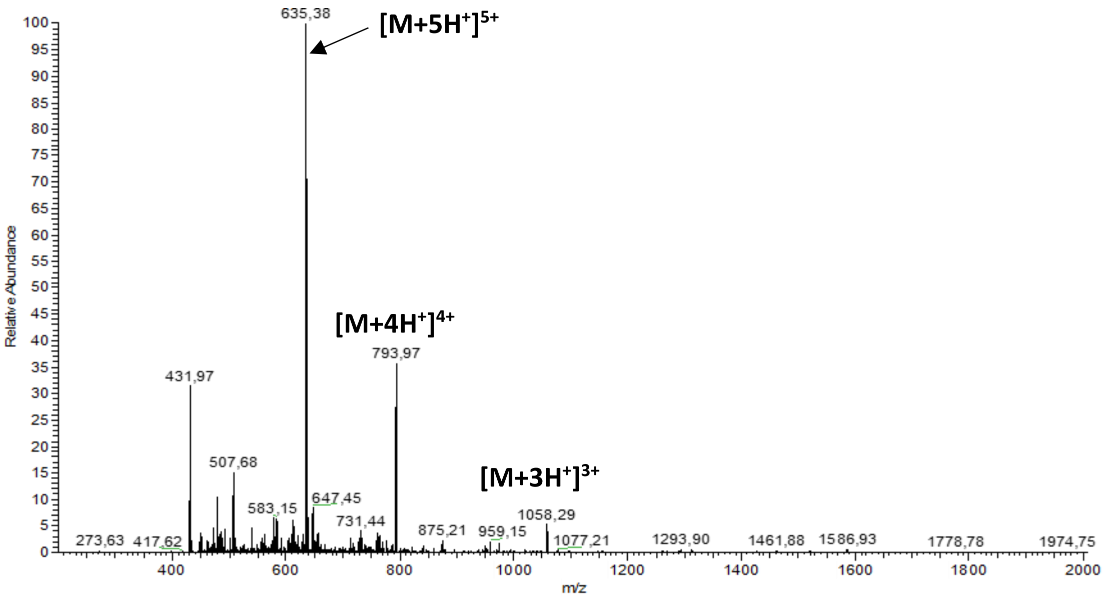

Figure S21 - ESI-IT MS spectrum (positive mode) of conjugate Cq-C4-Transportan (**5b**) (observed as the tri-, tetra-, and penta-protonated ions)

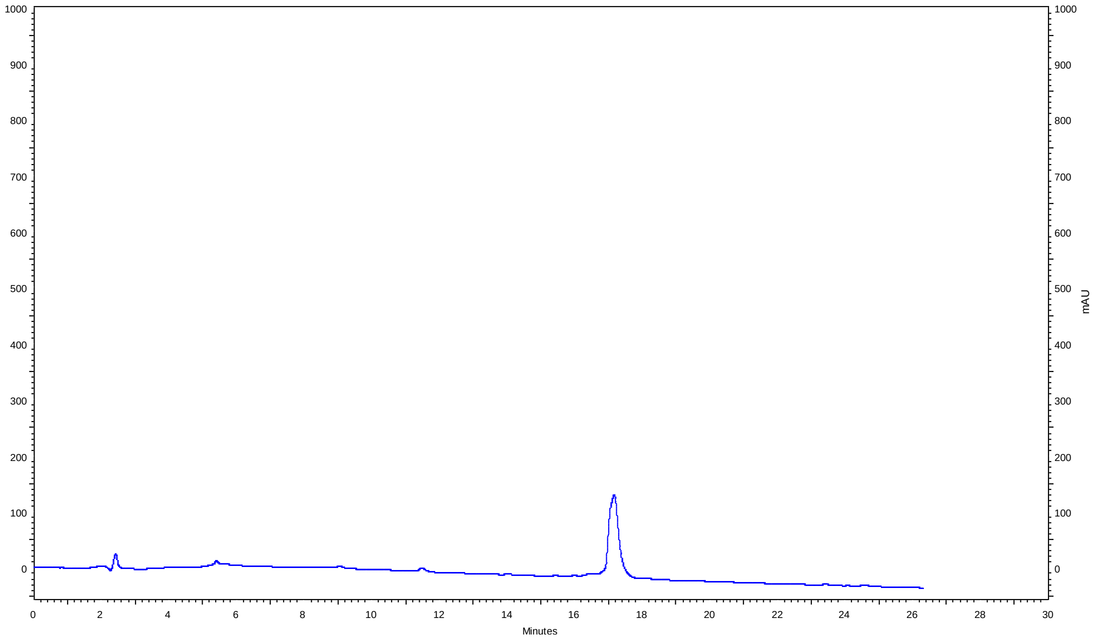

Figure S22 – Chromatogram obtained for Cq-C4-Transportan (**5b**), with a gradient elution of 0-100% ACN in water (0,05% TFA) in a RP-18E (5 μm), for 30 min and a flow rate of 1 ml/min, with detection at λ=220 nm.

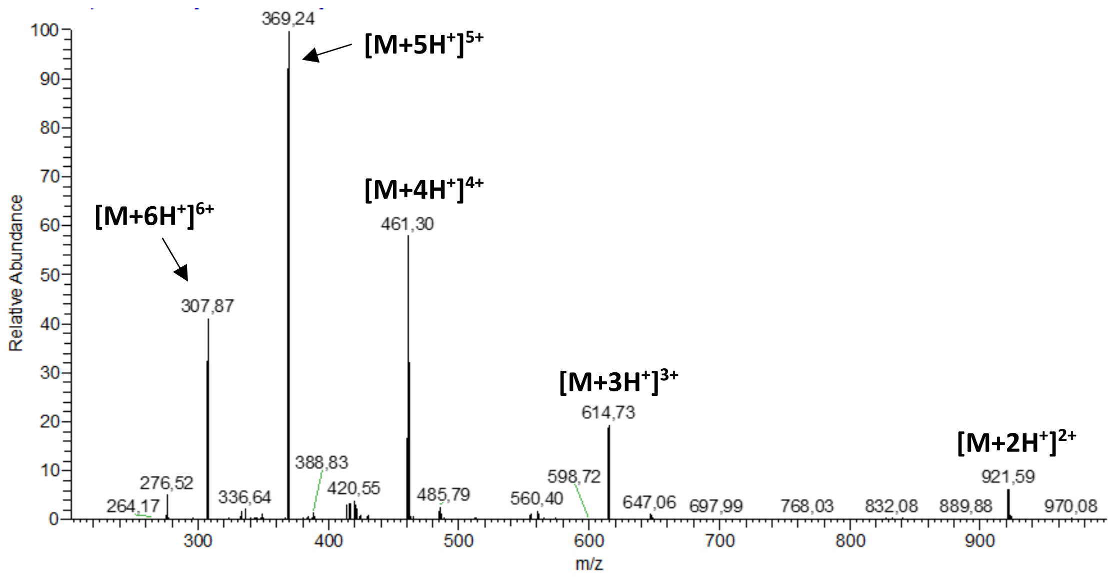

Figure S23 - ESI-IT MS spectrum (positive mode) of conjugate Cq-C4-DPT-sh1 (**5c**) (observed as the di-, tri-, tetra-, penta-, and hexa-protonated ions)

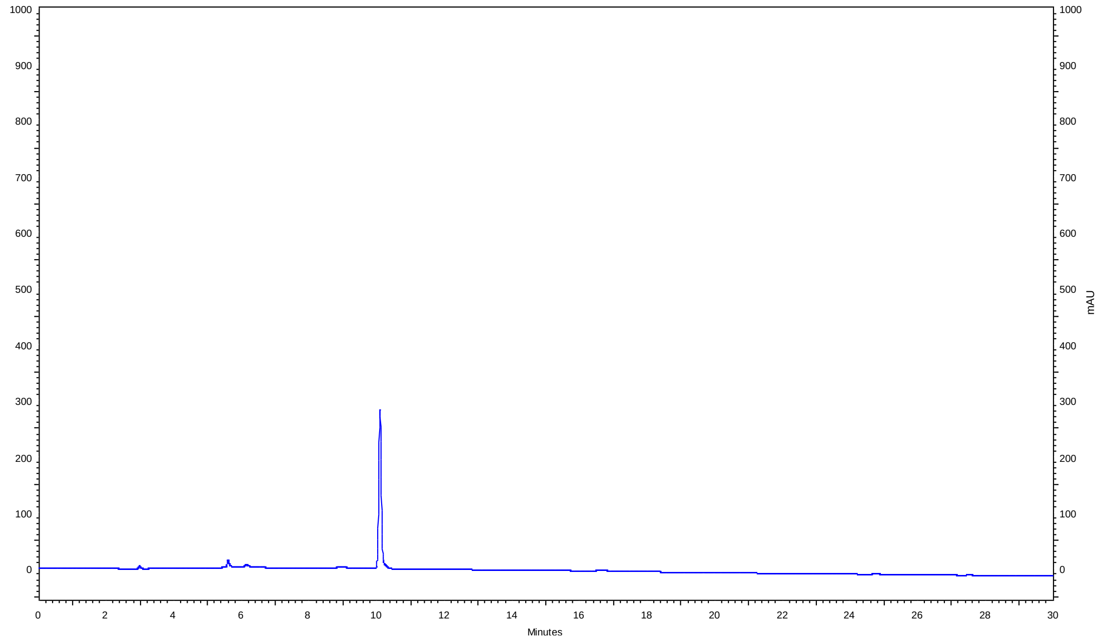

Figure S24– Chromatogram obtained for Cq-C4-DPT-sh1 (**5c**), with a gradient elution of 0-100% ACN in water (0,05% TFA) in a RP-18E (5 µm), for 30 min and a flow rate of 1 ml/min, with detection at λ=220 nm.

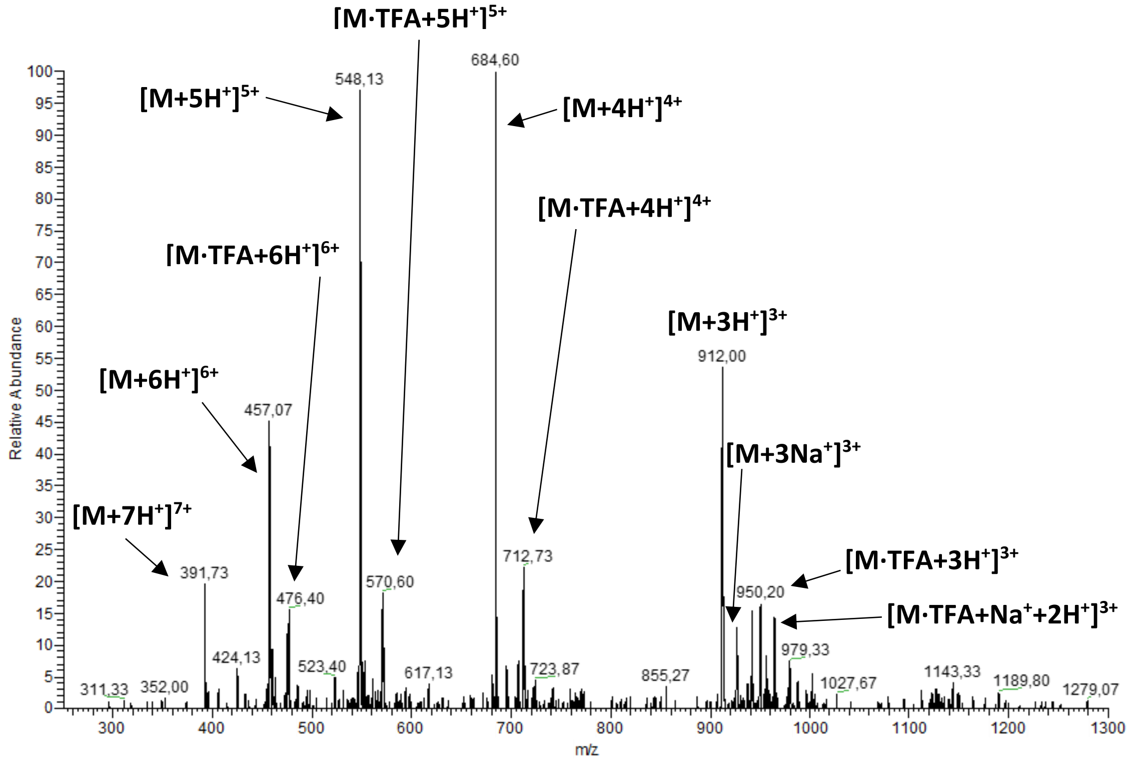

Figure S25 - ESI-IT MS spectrum (positive mode) of conjugate Cq-C4-DPT-sh2 (**5d**) (observed as the tri-, tetra-, penta-, hexa-, and hepta-protonated ions; sodium and TFA adducts at different protonation states also observed)

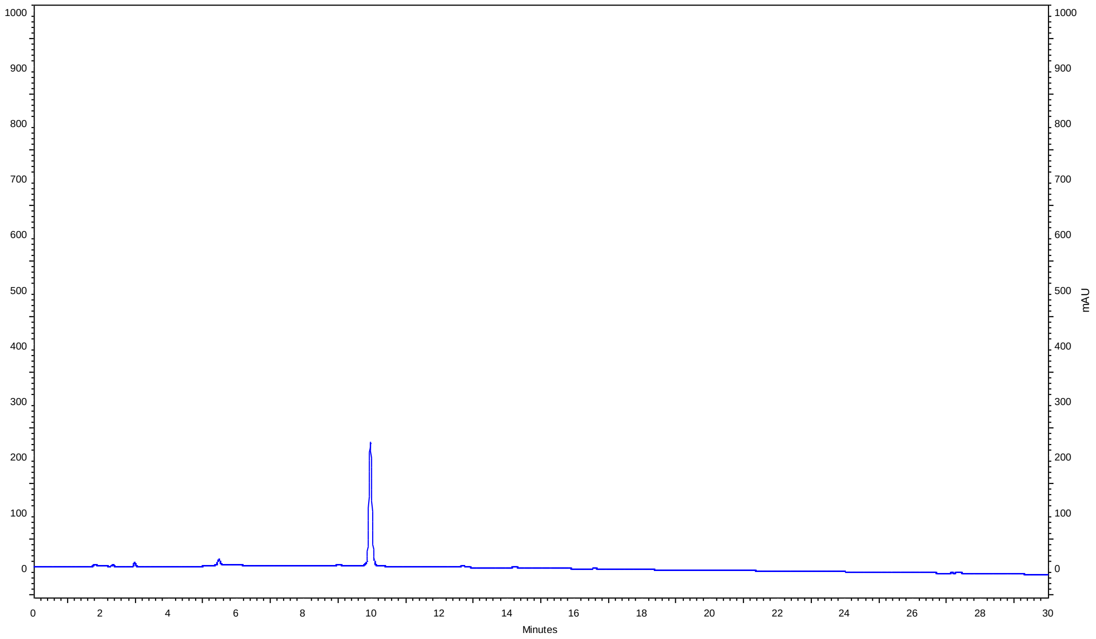

Figure S26 – Chromatogram obtained for Cq-C4-DPT-sh2 (**5d**), with a gradient elution of 0-100% ACN in water (0,05% TFA) in a RP-18E (5 μm), for 30 min and a flow rate of 1 ml/min, with detection at λ=220 nm.

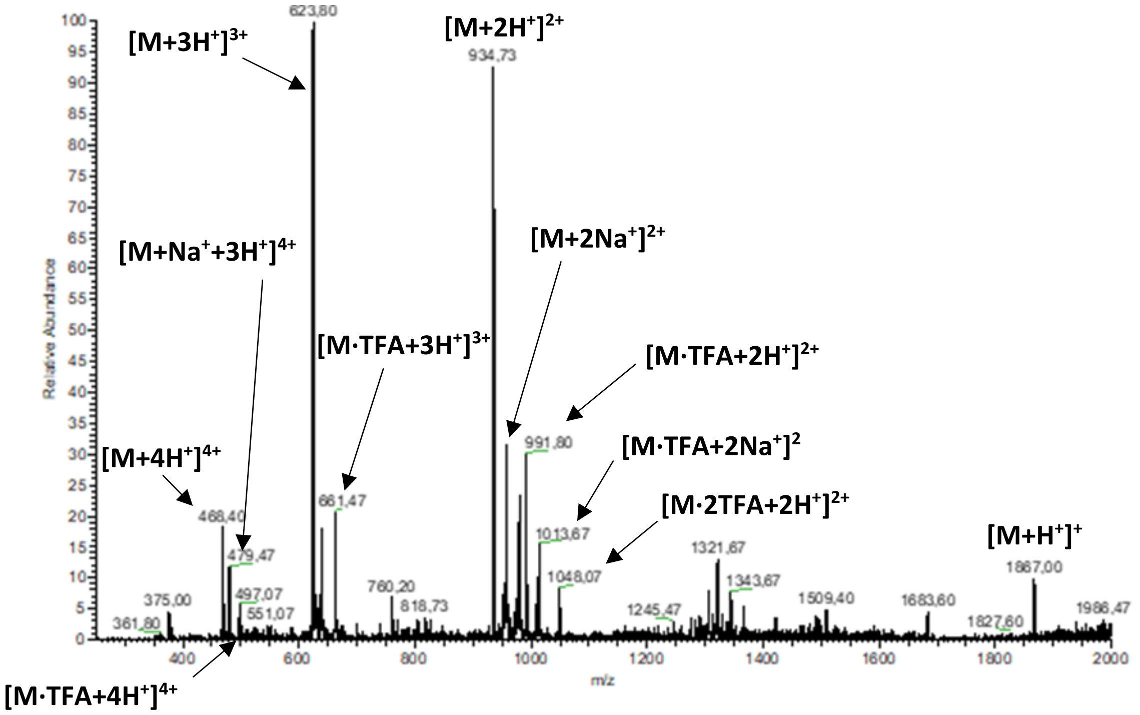

Figure S27 - ESI-IT MS spectrum (positive mode) of conjugate Cq-C4-IDR-1018 (**5e**) (observed as the quasi-molecular ion, and also as the di-, tri-, and tetra-protonated ions; sodium and TFA adducts at different protonation states also observed)

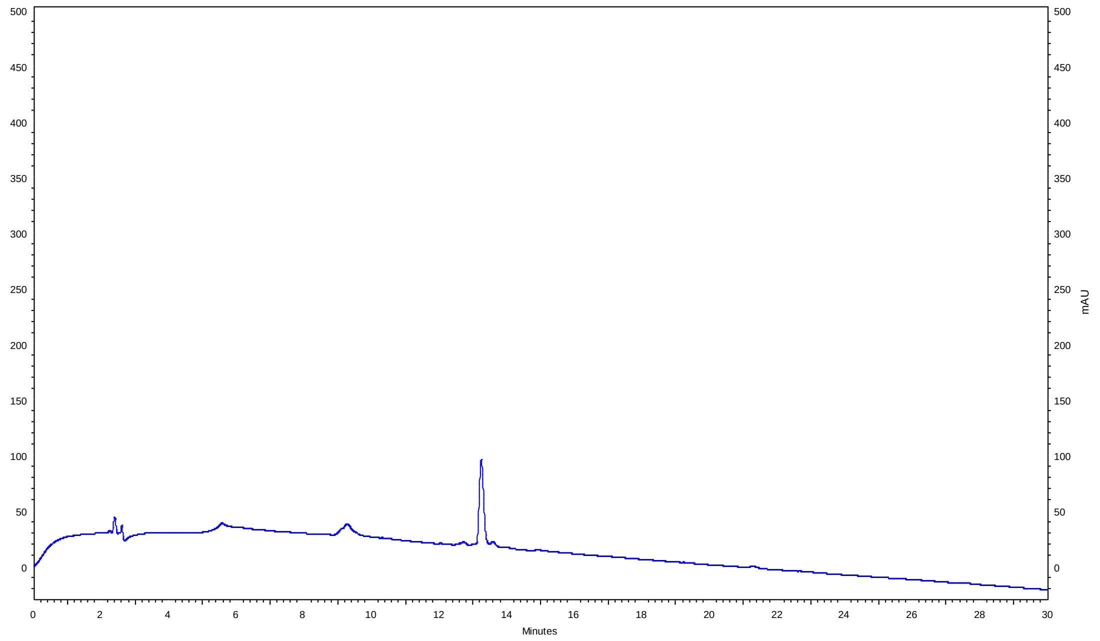

Figure S28 – Chromatogram obtained for Cq-C4-IDR-1018 (**5e**), with a gradient elution of 0-100% ACN in water (0,05% TFA) in a RP-18E (5 µm), for 30 min and a flow rate of 1 ml/min, with detection at λ=220 nm.

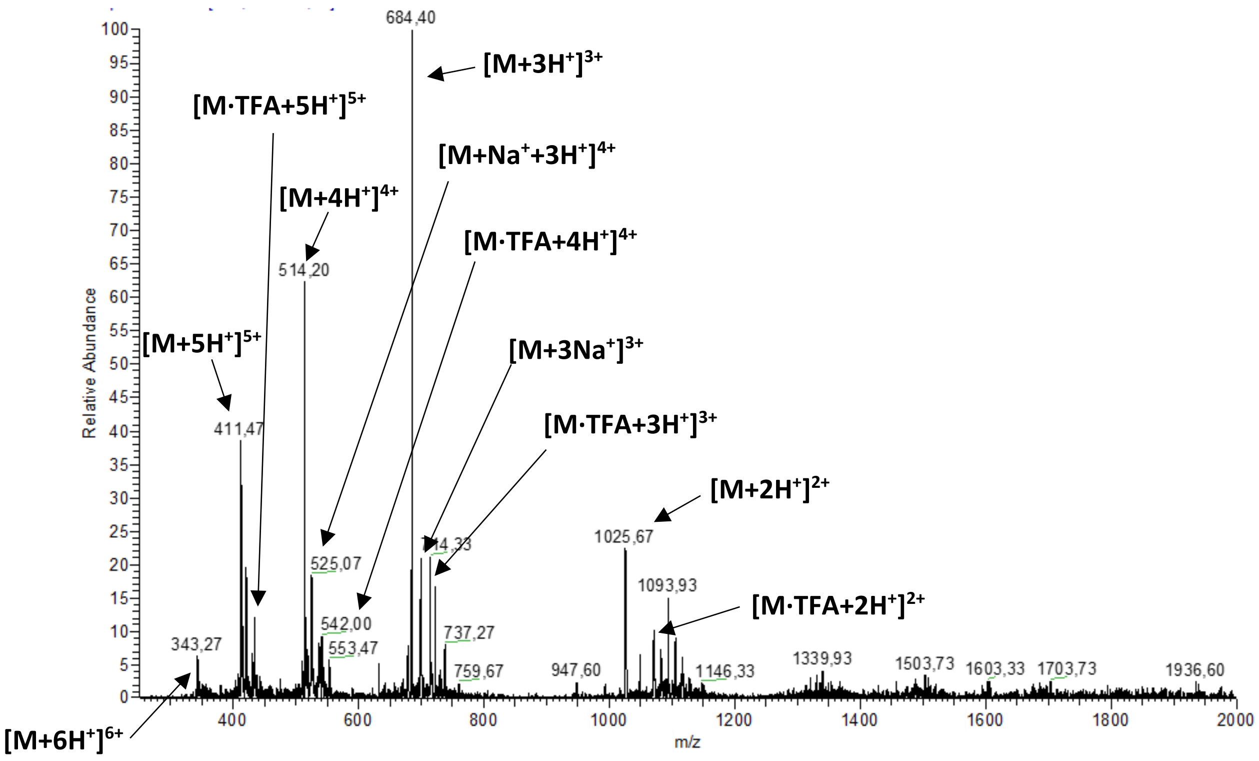

Figure S29 - ESI-IT MS spectrum (positive mode) of conjugate Cq-C4-TAT (**5f**) (observed as the di-, tri-, tetra-, penta-, and hexa-protonated ions; sodium and TFA adducts at different protonation states also observed)

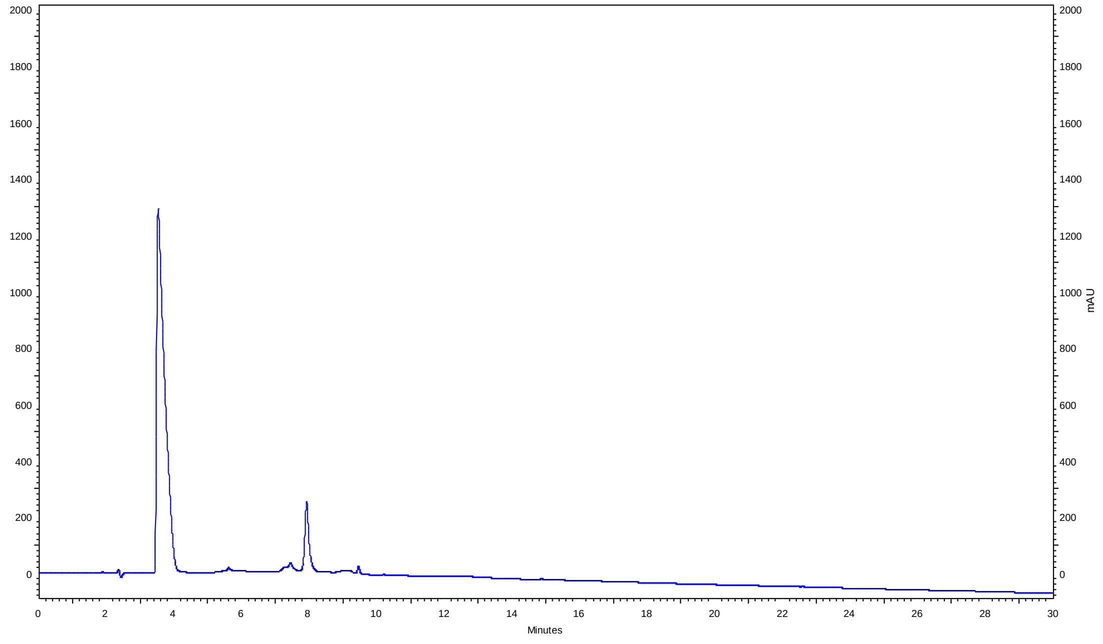

Figure S30 – Chromatogram obtained for Cq-C4-TAT (**5f**), with a gradient elution of 0-100% ACN in water (0,05% TFA) in a RP-18E (5 µm), for 30 min and a flow rate of 1 ml/min, with detection at λ=220 nm. The peak at RT ≈ 4 min is due to acetic acid, added to the samples for complete peptide solubilization.

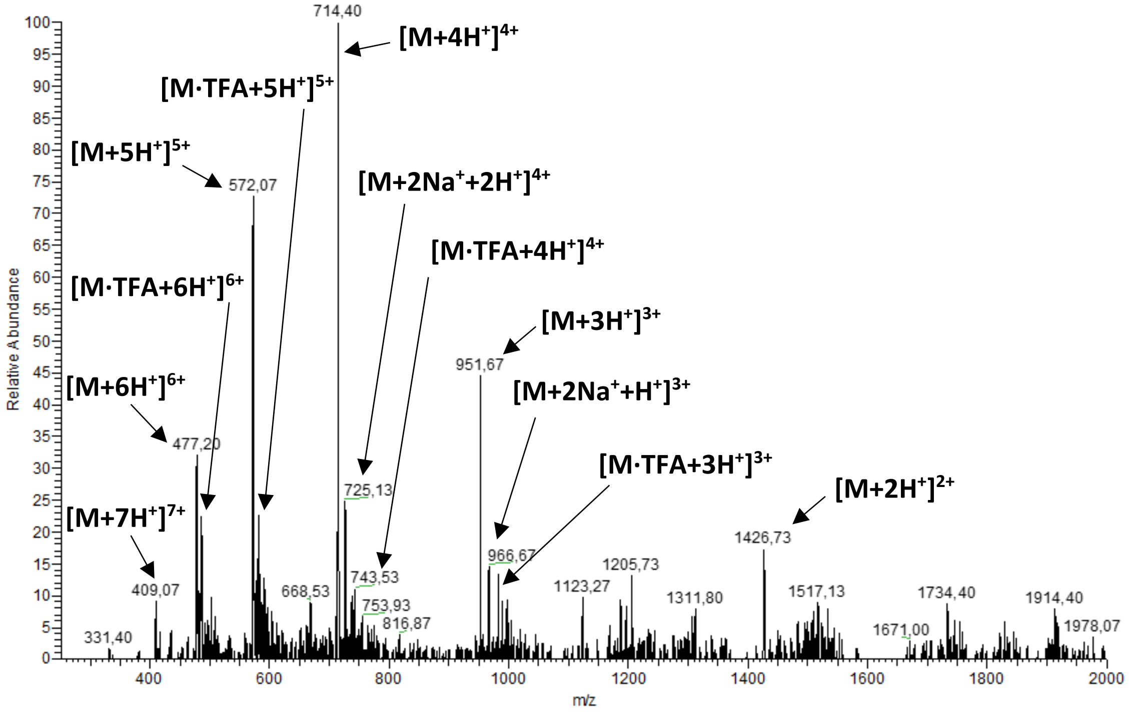

Figure S31 - ESI-IT MS spectrum (positive mode) of conjugate Cq-C4-PasTAT (**5g**)

(observed as the di-, tri-, tetra-, penta-, and hexa-protonated ions;

sodium and TFA adducts at different protonation states also observed)

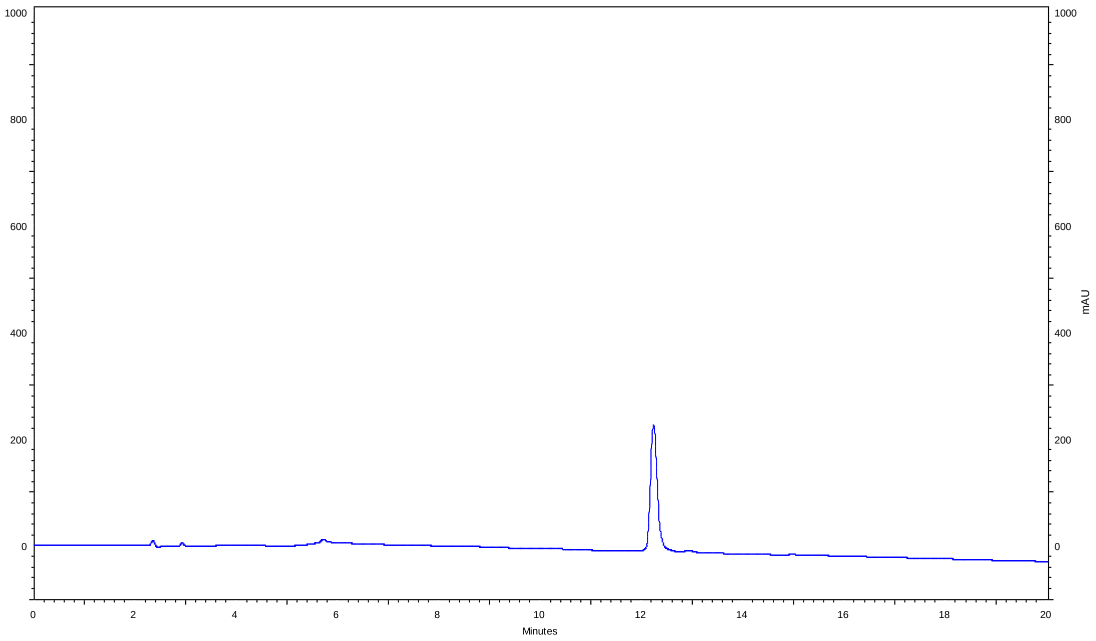

Figure S32 – Chromatogram obtained for Cq-C4-PasTAT (**5g**), with a gradient elution of 0-66% ACN in water (0,05% TFA) in a RP-18E (5 μm), for 20 min and a flow rate of 1 ml/min, with detection at λ=220 nm.

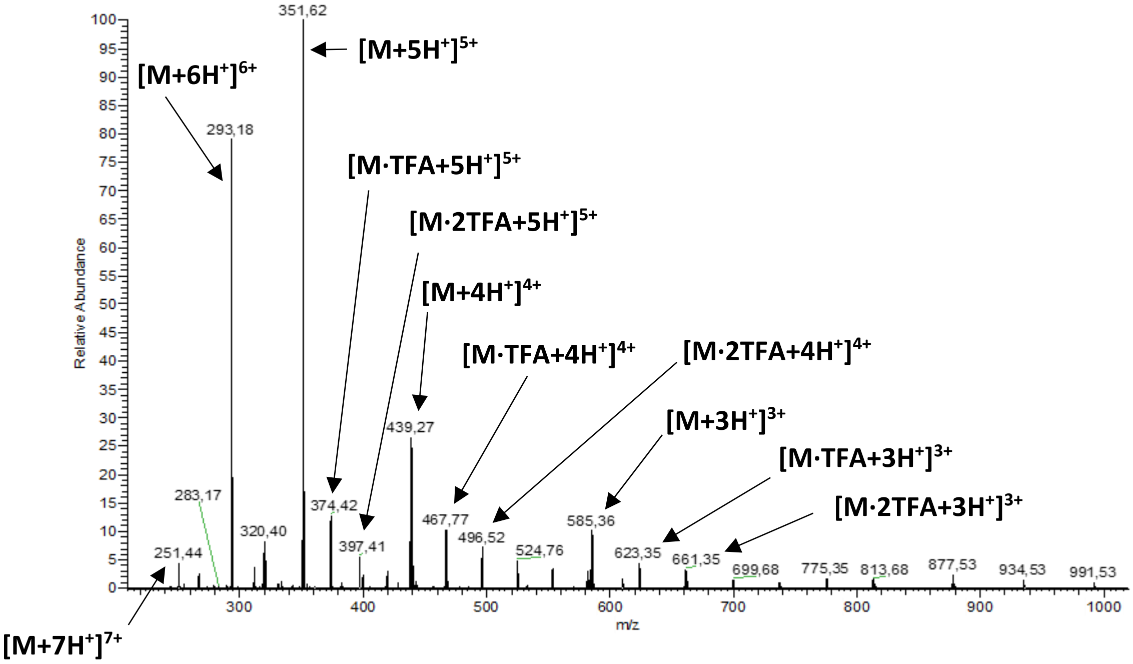

Figure S33 - ESI-IT MS spectrum (positive mode) of conjugate Cq-C4-R9 (**5h**)
(observed as tri-, tetra-, penta-, hexa-, and hepta-protonated ions;
TFA adducts at different protonation states also observed)

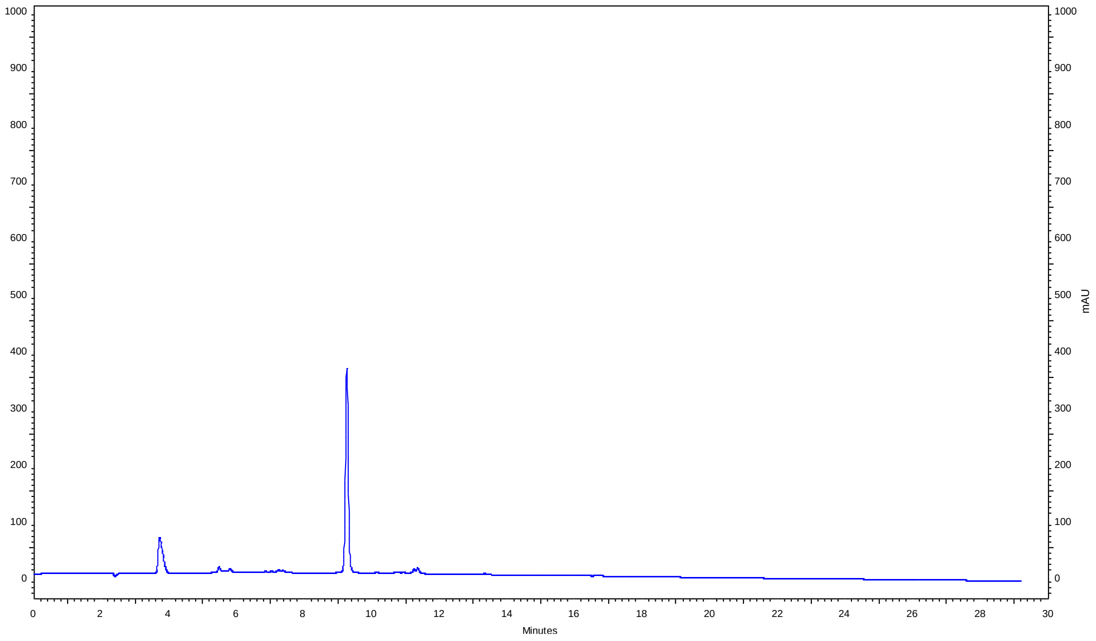

Figure S34 – Chromatogram obtained for Cq-C4-R9 (**5h**) after purification, with a gradient elution of 0-100% ACN in water (0,05% TFA) in a RP-18E (5 µm), for 30 min and a flow rate of 1 ml/min, with detection at λ=220 nm.

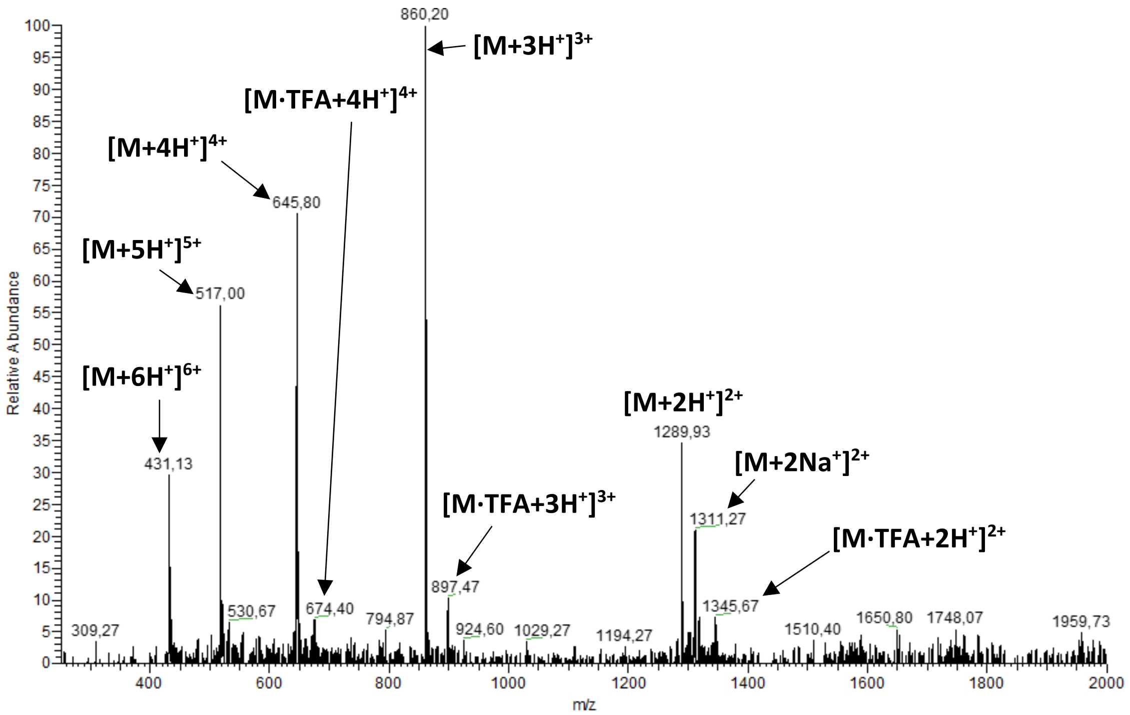

Figure S35 - ESI-IT MS spectrum (positive mode) of conjugate Cq-C4-Penetratin (**5i**) (observed as the di-, tri-, tetra-, penta-, and hexa-protonated ions; sodium and TFA adducts at different protonation states also observed)

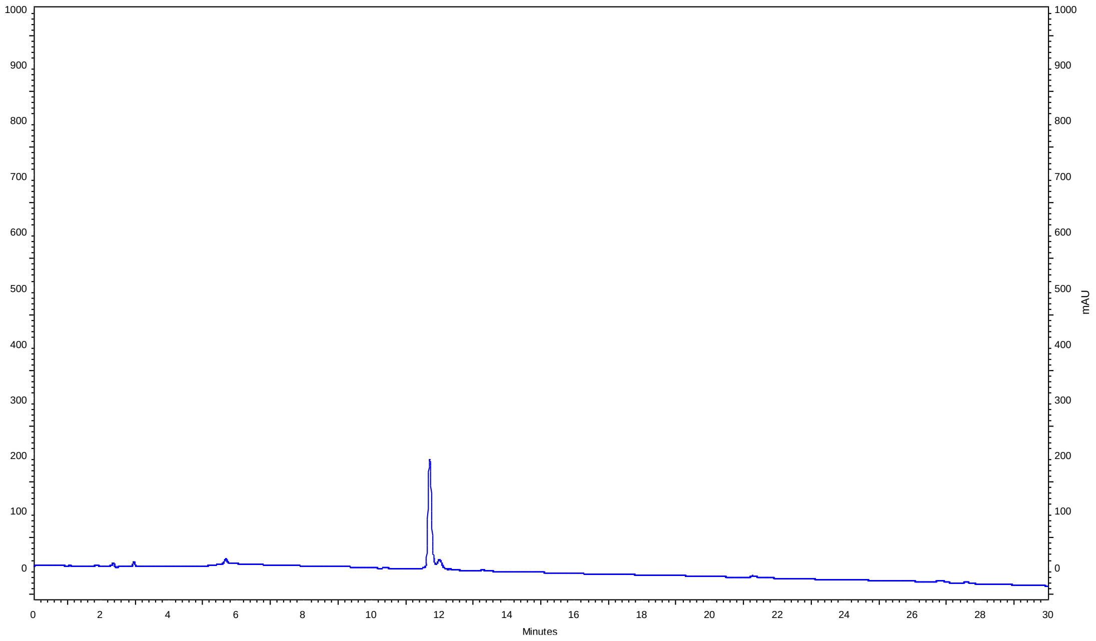

Figure S36 – Chromatogram obtained for Cq-C4-Penetratin (**5i**), with a gradient elution of 0-100% ACN in water (0,05% TFA) in a RP-18E (5 μm), for 30 min and a flow rate of 1 ml/min, with detection at λ=220 nm.

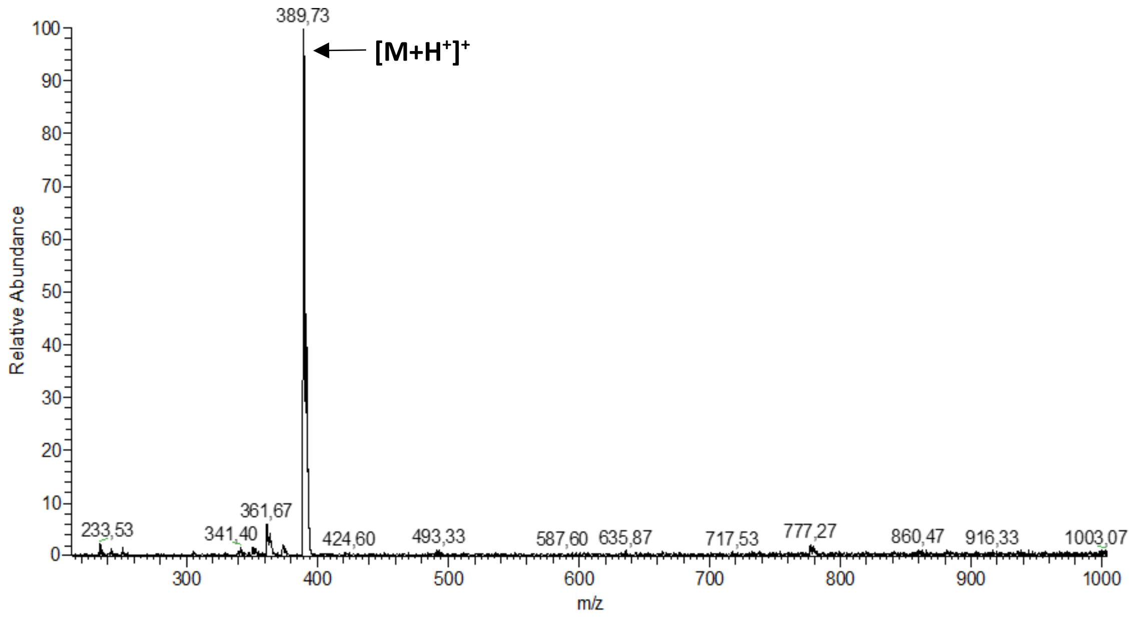

Figure S37 - ESI-IT MS spectrum (positive mode) of Cq-C6-N_3 (**7**)

(observed as the quasi-molecular ion)

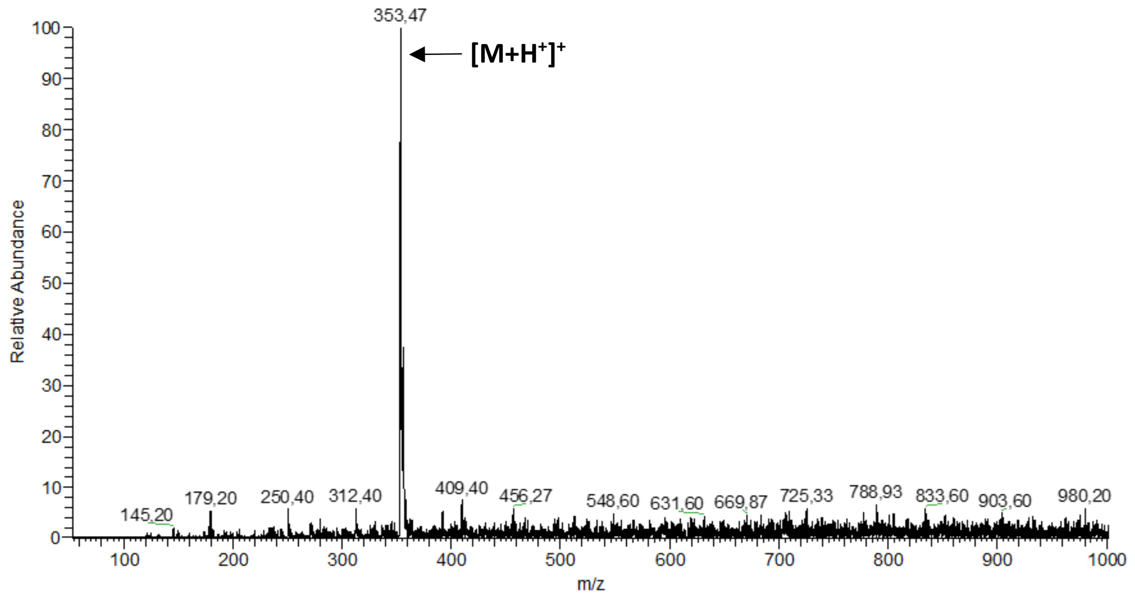

Figure S38 - ESI-IT MS spectrum (positive mode) of Cq-Cys (**8**)

(observed as the quasi-molecular ion)

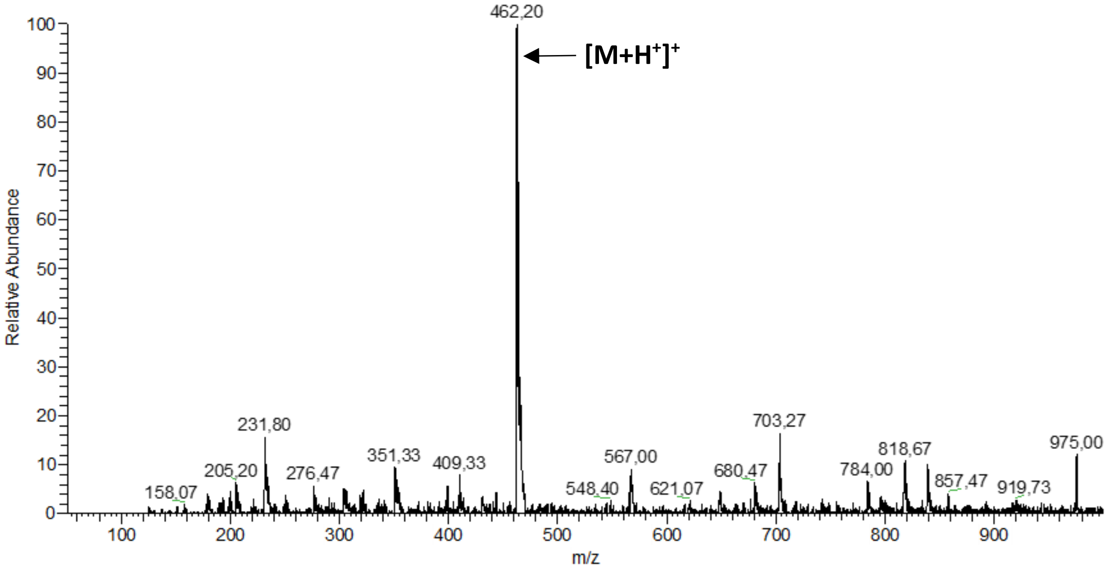

Figure S39 - ESI-IT MS spectrum (positive mode) of Cq-Cys(2-PDS) (**9**)

(observed as the quasi-molecular ion)

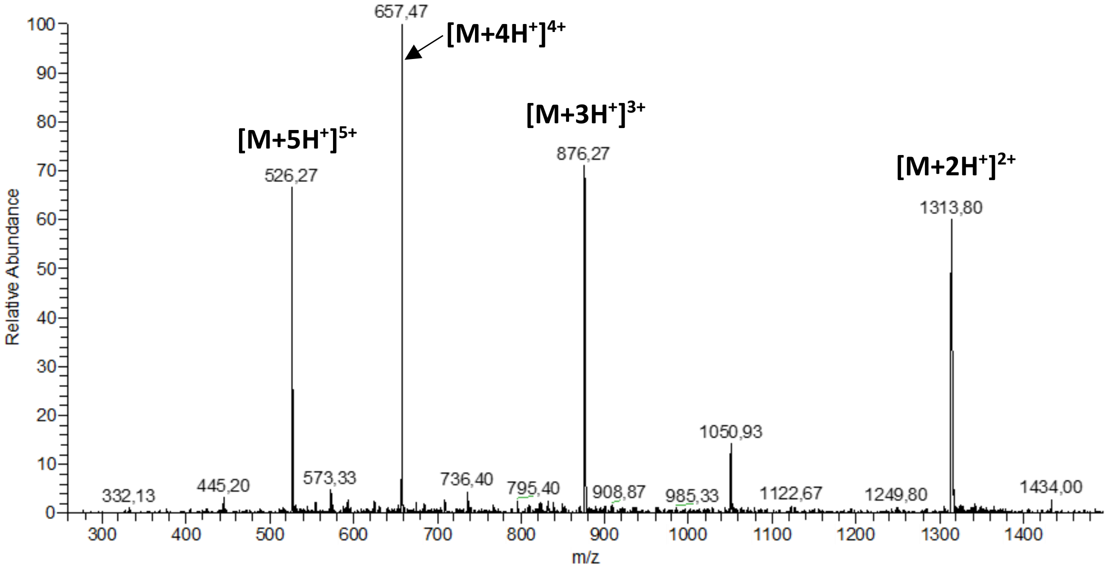

Figure S40 - ESI-IT MS spectrum (positive mode) of Cq-C10-TP10 (**10**)

(observed as the di-, tri-, tetra-, and penta-protonated ions)

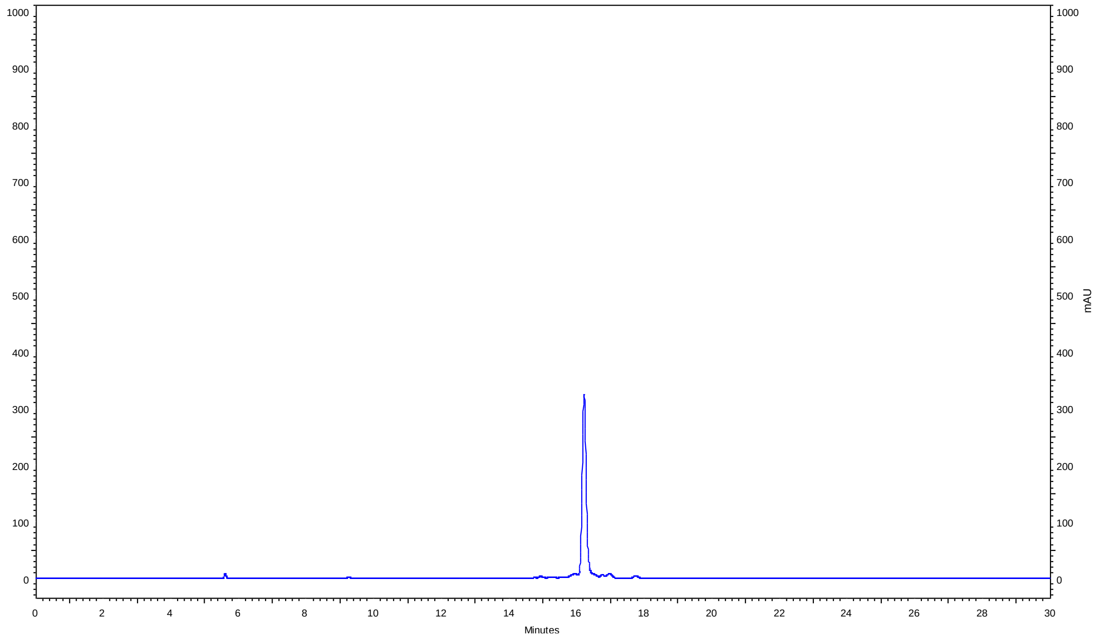

Figure S41 – Chromatogram obtained for Cq-C10-TP10 (**10**), with a gradient elution of 0-100% ACN in water (0.05% TFA) in a RP-18E (5 μm), for 30 min and a flow rate of 1 ml/min, with detection at λ=220 nm.

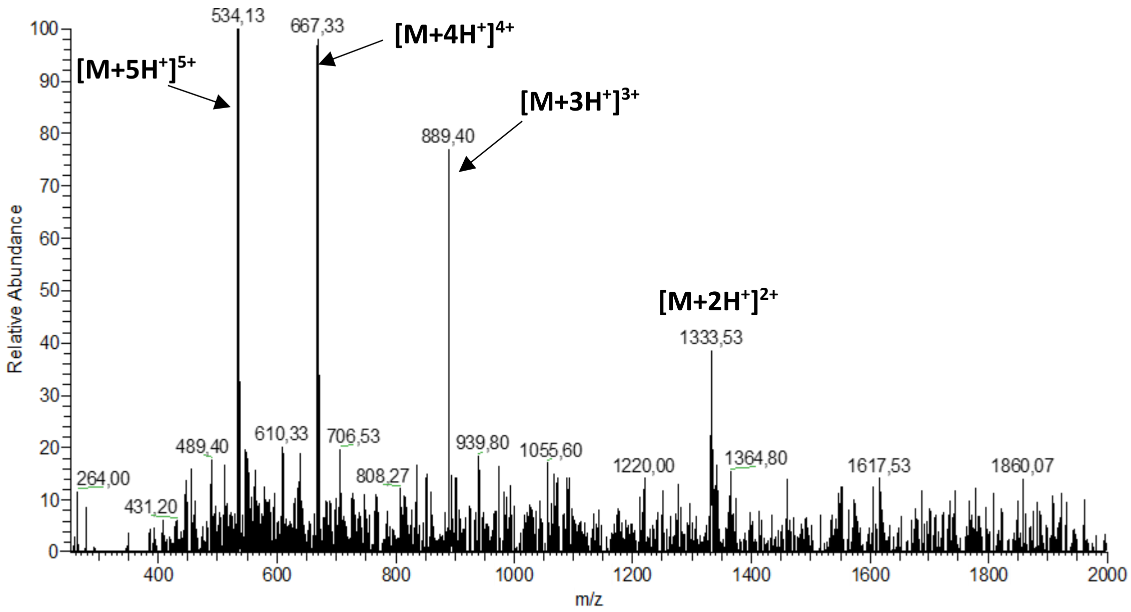

Figure S42 - ESI-IT MS spectrum (positive mode) obtained for Cq-TR-TP10 (**11**) (observed as the di-, tri, tetra-, and penta-protonated ions)

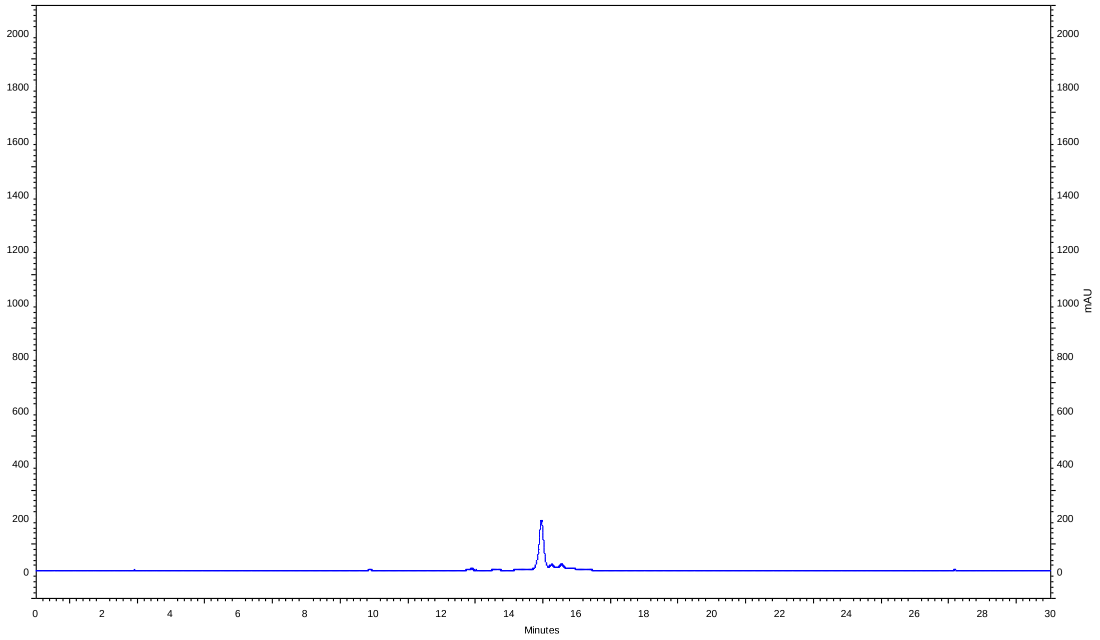

Figure S43 – Chromatogram obtained for Cq-TR-TP10 (**11**), with a gradient elution of 0-100% ACN in water (0.05% TFA) in a RP-18E (5 μm), for 30 min and a flow rate of 1 ml/min, with detection at λ=220 nm.

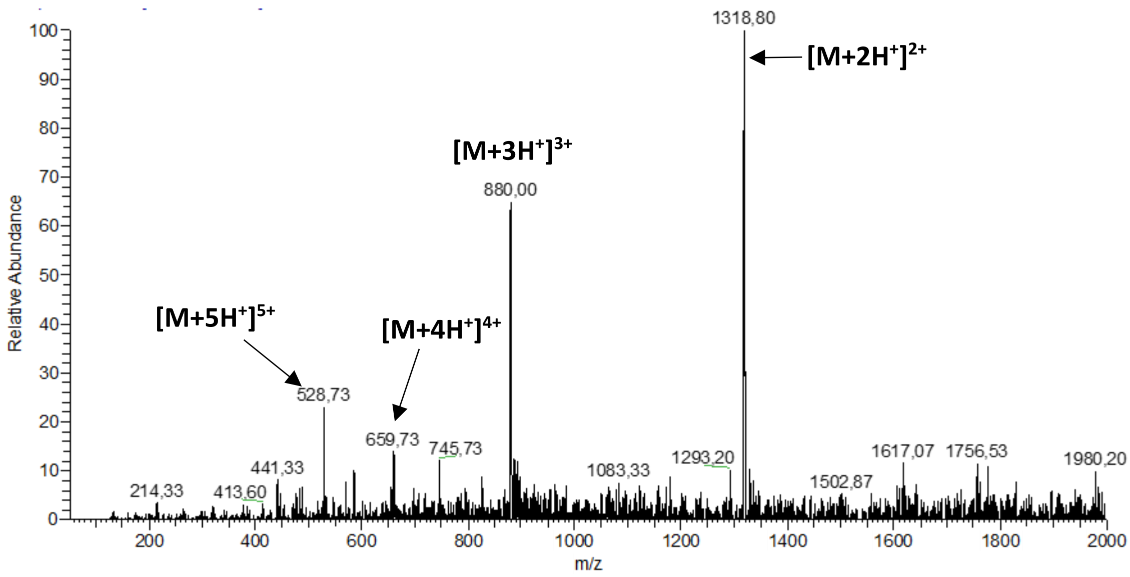

Figure S44 - ESI-IT MS spectrum (positive mode) obtained for Cq-S-S-TP10 (**12**) (observed as the di-, tri-, tetra-, and penta-protonated ions)

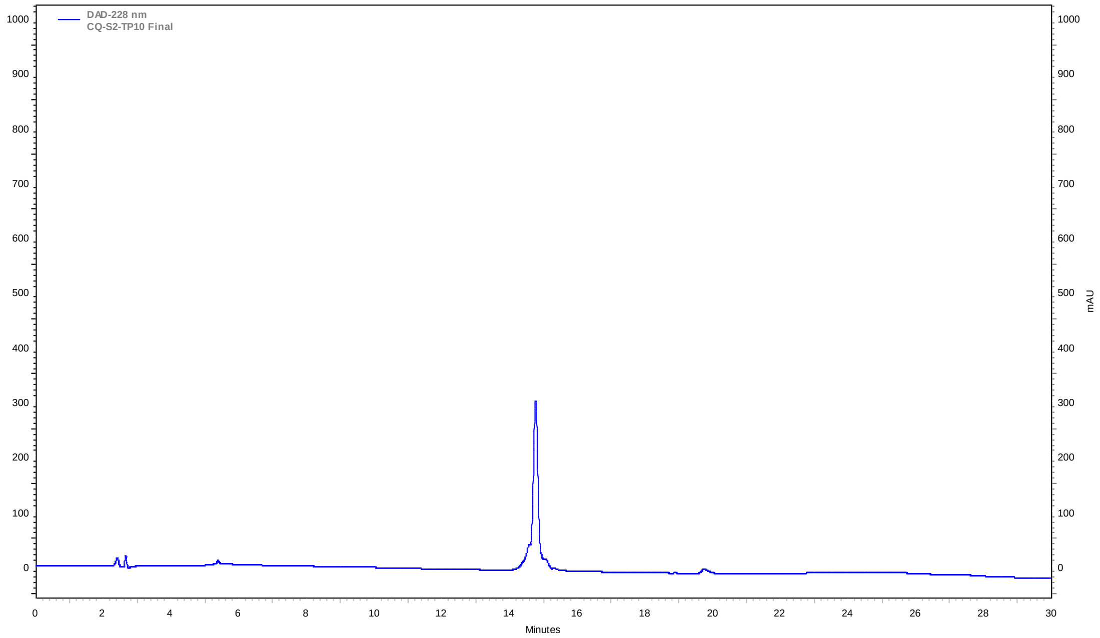

Figure S45 – Chromatogram obtained for CQn-S-S-TP10 (**12**), with a gradient elution of 0-100% ACN in water (0.05% TFA) in a RP-18E (5 μm), for 30 min and a flow rate of 1 ml/min, with detection at λ=220 nm.

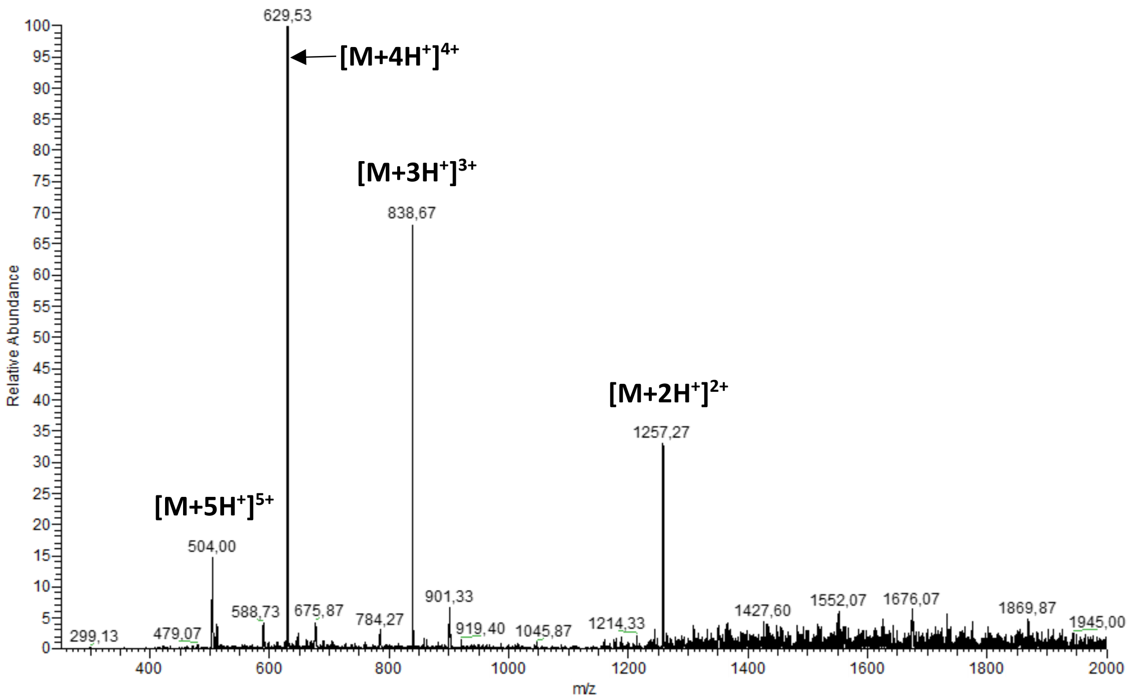

Figure S46 - ESI-IT MS spectrum (positive mode) of TP10-C4-Cq (**13**)

(observed as the di-, tri-, tetra-, and penta-protonated ions)

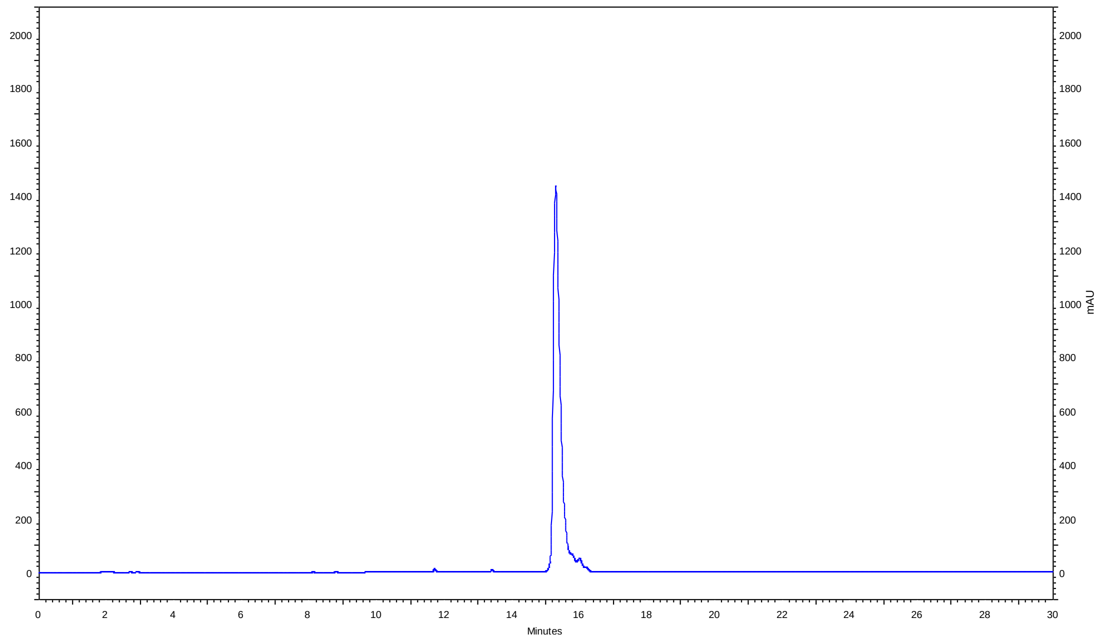

Figure S47 - Chromatogram obtained for TP10-C4-Cq (**13**), with a gradient elution of 0-100% ACN in water (0.05% TFA) in a RP-18E (5 µm), for 30 min and a flow rate of 1 ml/min, with detection at λ=220 nm.

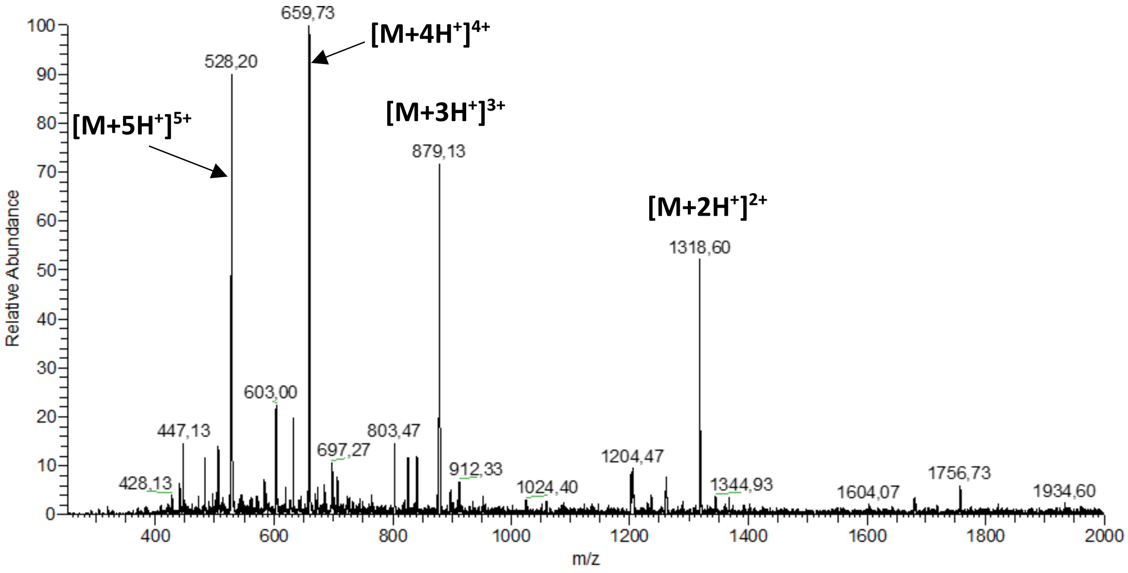

Figure S48 - ESI-IT MS spectrum (positive mode) of TP10-S-S-Cq (**14**) (observed as the di-, tri-, tetra-, and penta-protonated ions)

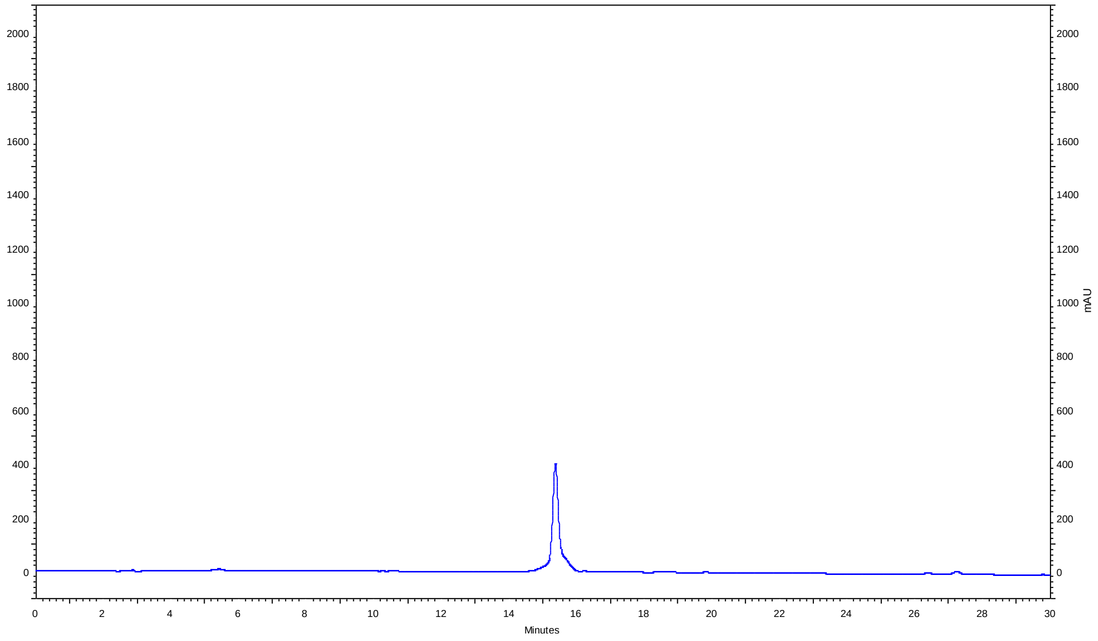

Figure S49 - Chromatogram obtained for TP10-S-S-Cq (**14**), with a gradient elution of 0-100% ACN in water (0.05% TFA) in a RP-18E (5 μm), for 30 min and a flow rate of 1 ml/min, with detection at λ=220 nm.

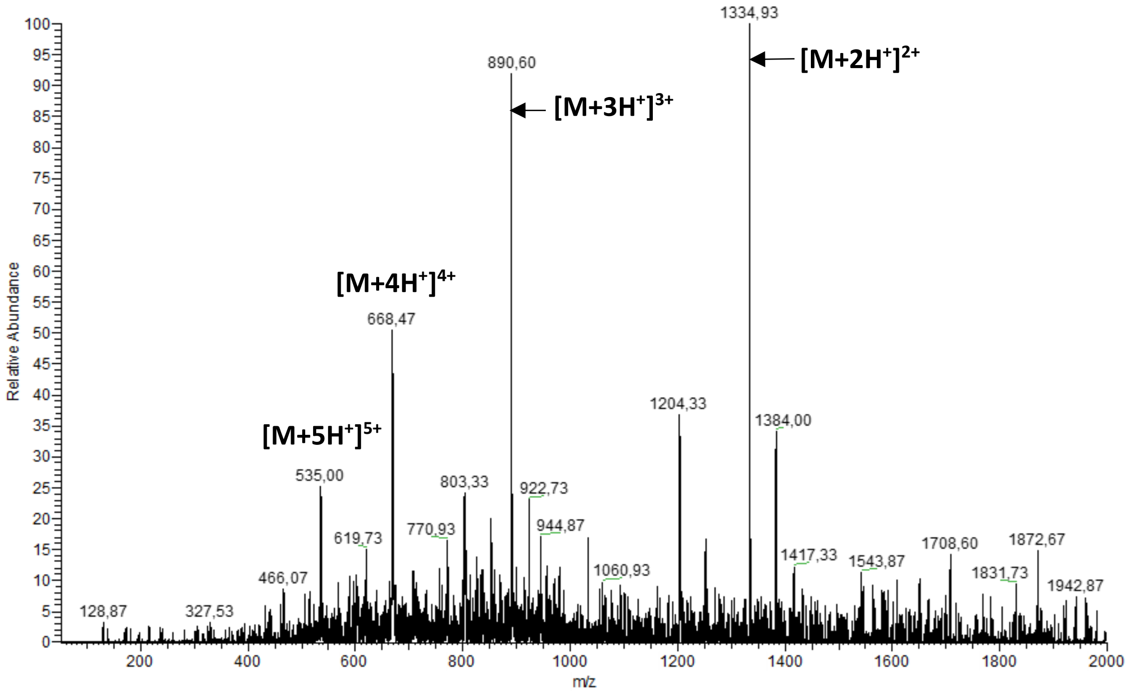

Figure S50 - ESI-IT MS spectrum (positive mode) of TP10-K(CF) (**15**) (observed as the di-, tri-, tetra-, and penta-protonated ions)

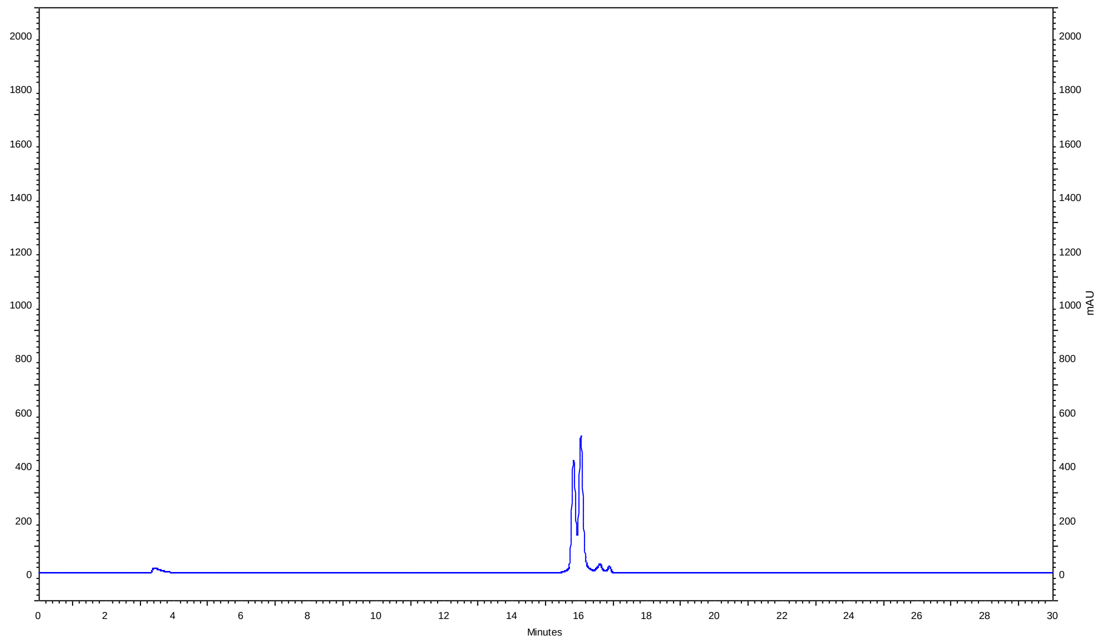

Figure S51 – Chromatogram obtained for TP10-K(CF) (**15**), with a gradient elution of 0-100% ACN in water (0.05% TFA) in a RP-18E (5 µm), for 30 min and a flow rate of 1 ml/min, with detection at λ=220 nm. Note: a mixture of 5- and 6-carboxyfluorescein was used to label the peptide, hence the two peaks observed in the HPLC trace.

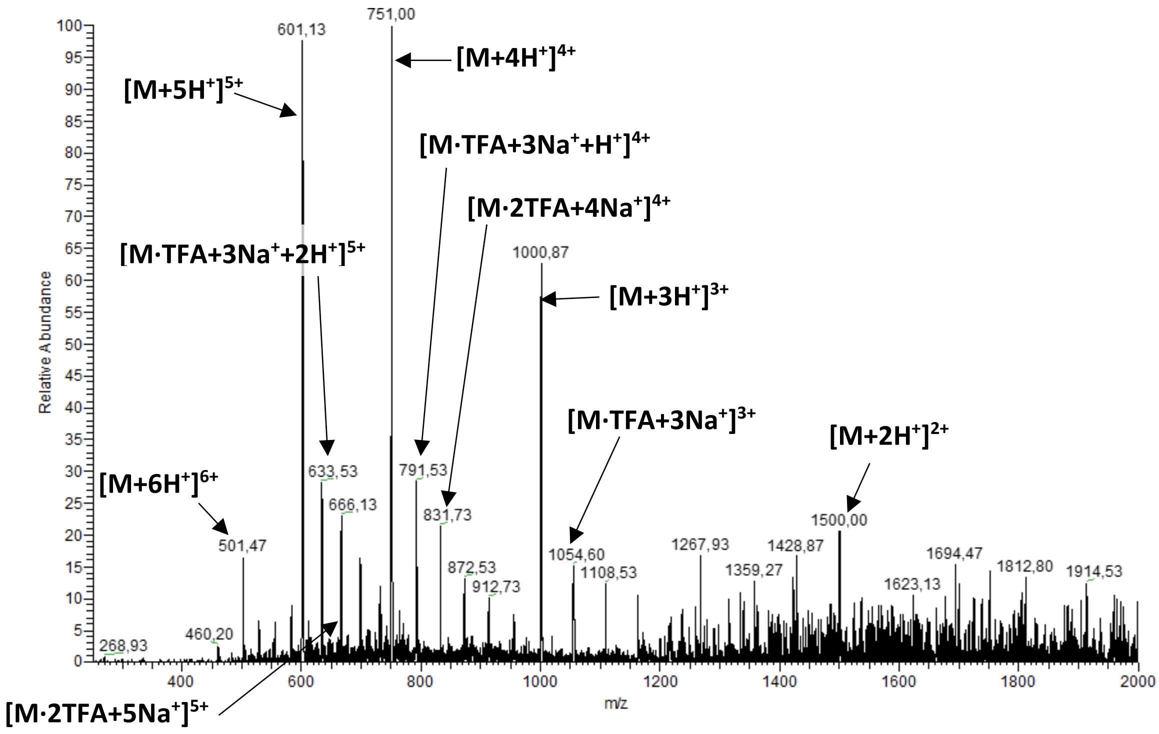

Figure S52 - ESI-IT MS spectrum (positive mode) of Cq-C4-TP10-K(CF) (**16**) (observed as the di-, tri-, tetra-, penta-, and hexa-protonated ions; sodium and TFA adducts at different protonation states also observed)

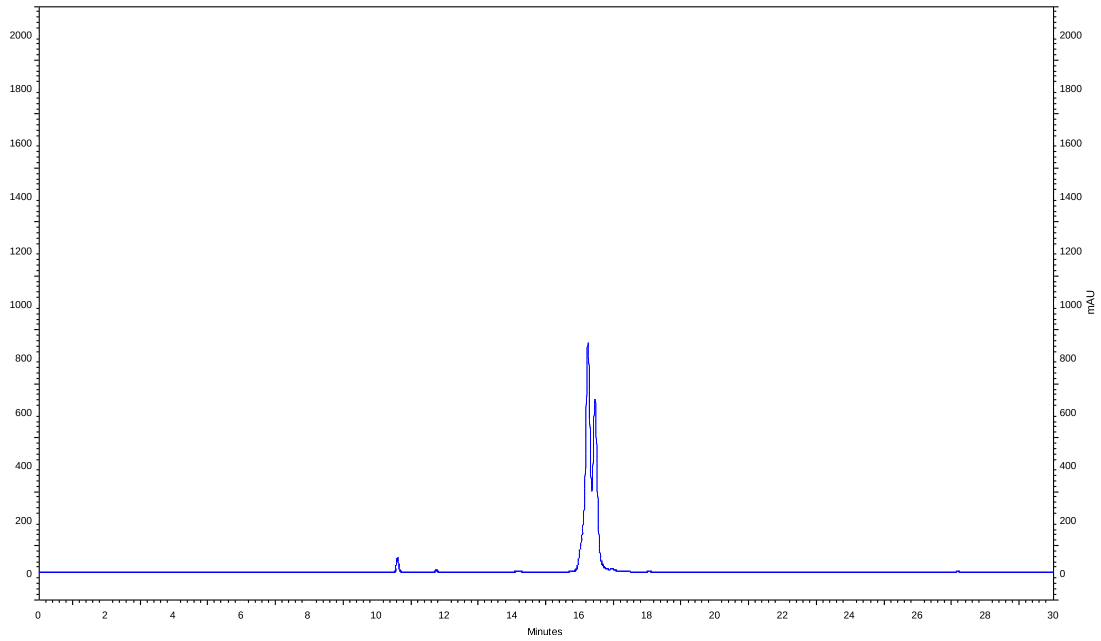

Figure S53 – Chromatogram for Cq-C4-TP10-K(CF) (**16**), with a gradient elution of 0-100% ACN in water (0.05% TFA) in a RP-18E (5 μm), for 30 min and a flow rate of 1 ml/min, with detection at λ=220 nm. Note: a mixture of 5- and 6-carboxyfluorescein was used to label the peptide, hence the two peaks observed in the HPLC trace.

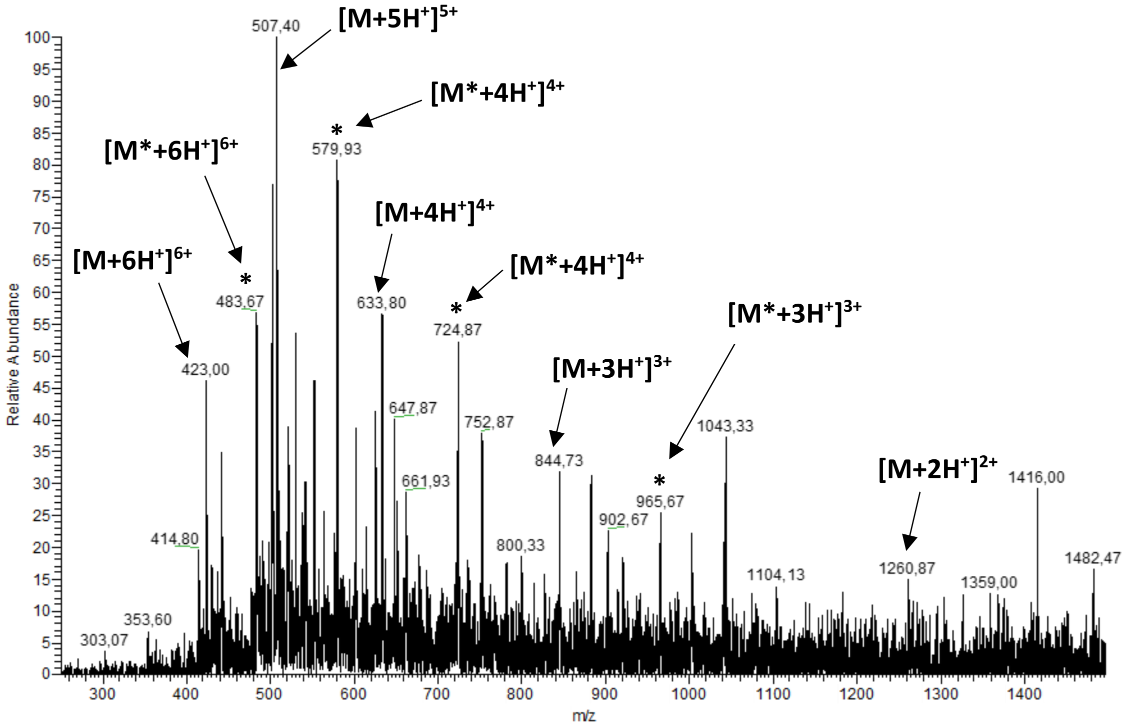

Figure S54 - ESI-IT MS spectrum (positive mode) obtained for crude Cq-C4-TAT-K(CF) (**17**) (observed as the di-, tri-, tetra-, penta-, and hexa-protonated ion; peaks labeled with an asterisk are due to a doubly CF-labeled conjugate that was removed upon purification, see HPLC trace below)

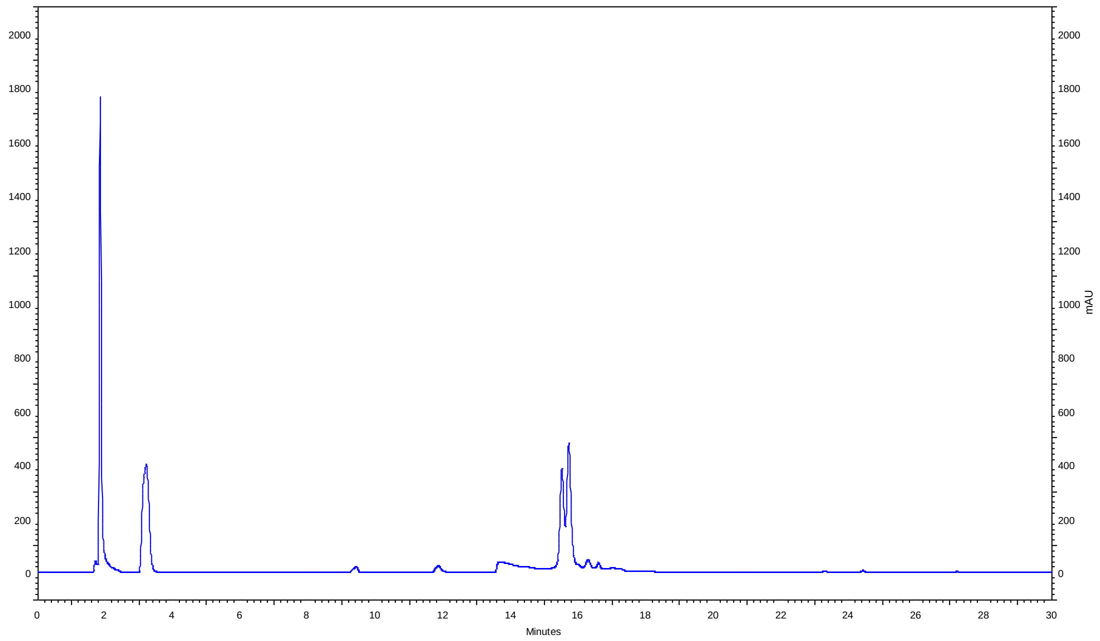

Figure S55 – Chromatogram obtained for Cq-C4-TAT-K(CF) (**17**), with a gradient elution of 0-100% ACN in water (0.05% TFA) in a RP-18E (5 μm), for 30 min and a flow rate of 1 ml/min, with detection at λ=220 nm. The peak at RT ≈ 3 min is due to acetic acid, added to the samples for complete peptide

solubilization. Note: a mixture of 5- and 6-carboxyfluorescein was used to label the peptide, hence the two peaks observed in the HPLC trace.
